# Material and shape perception based on two types of intensity gradient information

**DOI:** 10.1371/journal.pcbi.1006061

**Published:** 2018-04-27

**Authors:** Masataka Sawayama, Shin'ya Nishida

**Affiliations:** Human Information Science Laboratory, NTT Communication Science Laboratories, Nippon Telegraph and Telephone Corporation, Atsugi, Kanagawa, Japan; Technische Universitat Chemnitz, GERMANY

## Abstract

Visual estimation of the material and shape of an object from a single image includes a hard ill-posed computational problem. However, in our daily life we feel we can estimate both reasonably well. The neural computation underlying this ability remains poorly understood. Here we propose that the human visual system uses different aspects of object images to separately estimate the contributions of the material and shape. Specifically, material perception relies mainly on the intensity gradient magnitude information, while shape perception relies mainly on the intensity gradient order information. A clue to this hypothesis was provided by the observation that luminance-histogram manipulation, which changes luminance gradient magnitudes but not the luminance-order map, effectively alters the material appearance but not the shape of an object. In agreement with this observation, we found that the simulated physical material changes do not significantly affect the intensity order information. A series of psychophysical experiments further indicate that human surface shape perception is robust against intensity manipulations provided they do not disturb the intensity order information. In addition, we show that the two types of gradient information can be utilized for the discrimination of albedo changes from highlights. These findings suggest that the visual system relies on these diagnostic image features to estimate physical properties in a distal world.

## Author summary

Objects in our visual world contain a variety of material information. Although such information enables us to experience rich material impressions, it can be a distraction for the estimation of other physical properties such as shapes, albedos, and illuminations. The coupled estimation of these properties we humans perform in daily situations is one of the fundamental mysteries in visual neuroscience. Here, we show that material and shape perception depend on two different types of intensity gradient information. Specifically, our image analyses and psychophysical experiments show that a human’s material perception relies mainly on the intensity gradient magnitude information, while shape perception relies mainly on the intensity gradient order information. In addition, we show that the intensity order information can be utilized for discriminating albedo changes on an object surface from other physical properties including specular highlights.

## Introduction

The physical parameters that affect a retinal image are extremely complex. In addition, the same retinal image can be produced from an infinite number of combinations of materials, shapes, and illuminations in the distal world. Therefore, it is a hard ill-posed problem to estimate what exists in the distal world from a single retinal image. This appears to be a chicken-and-egg problem as material estimation requires knowledge about shape (and illumination), while shape estimation requires knowledge about material (and illumination). Nevertheless, (we believe) we can estimate the physical parameters that produce a retinal image. For instance, from a single photograph wherein a metal teapot is placed on a table, we can simultaneously judge the material and shape of the object. This paper concerns the visual processing underlying such simultaneous estimation.

**Fig 1:**
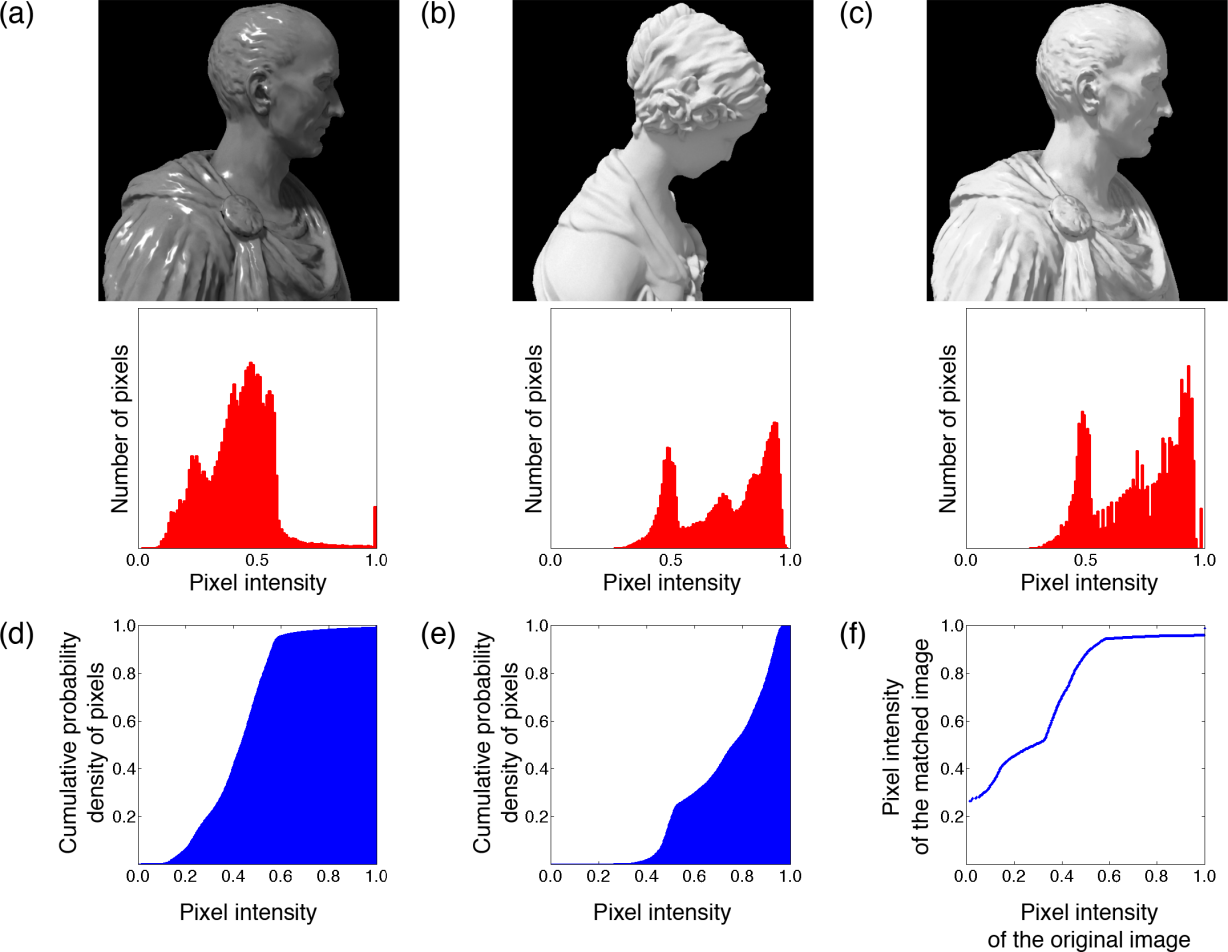
A histogram-matching method. The intensity histogram of the original image (a) is matched with that of the reference image (b) in the histogram-matched image (c). The geometric model of (a) is "Julius Caesar" designed by Yousef Mansy (https://pinshape.com/items/25809-3d-printed-julius-caesar-scan-the-world), and that of (b) is "Venus sculpture" designed by SHINING 3D (https://pinshape.com/items/19446-3d-printed-venus-sculpture). The cumulative probability densities of pixels of the original and reference image are shown in (d) and (e), respectively. (f) Pixel intensity of the histogram-matched image plotted as a function of that of the original image.

We found a clue for solving this problem in the image-based material editing methods developed in the computer graphics community [1–3]. By changing image parameters, not physical ones in the distal world, these methods can alter the material appearance of an object without significantly affecting its apparent shape or illumination. Among them, a simple yet effective method is to modulate the luminance histogram of an image. For instance, when the histogram of the original image in Fig 1(A) is matched with that of the reference image in Fig 1(B), the material appearance of the original image becomes very similar to that of the reference image (Fig 1(C)). Another example is the use of monotonic nonlinear tone-remapping for print or screen display devices to transform the intensity histogram of an input image and modify its qualitative appearance [4,5].

Successful manipulation of material appearance by histogram transformation suggests that luminance histograms contain critical information for material perception [6–12]. Specifically, Motoyoshi et al. [8] found that a surface tends to look glossier when the luminance histogram of the surface’s image is positively skewed. Although histogram skewness can be affected by various image parameters, it can be a very good predictor of apparent gloss when the images are nearly the same in other respects, as is the case for histogram-transformed images. Motoyoshi et al. [8] also showed that adaptation to textures with skewed statistics alters the perceived glossiness of surfaces subsequently viewed. These findings led them to conclude that human observers may use histogram skewness, or some image features correlated with it, in making judgments about glossiness.

However, when the spatial structure of an image is inconsistent with a natural glossy surface (e.g., a pixel- or phase-scrambled image), the image does not look glossy regardless of histogram manipulation [8]. Kim, Marlow and Anderson [13] further investigated spatial conditions of gloss perception and found that when specular highlights of an object image are inconsistent in position and/or orientation with the diffuse shading component, they look more like white blobs produced by surface reflectance changes (see also, [14–17]). Marlow, Todorovic and Anderson [18] have demonstrated that three-dimensional shape perception of a surface affects gloss perception of the surface.

These findings suggest that the visual system has to simultaneously solve at least three mutually dependent problems: it has to estimate surface material, surface shape (surface orientation), and reflectance changes. As mentioned above, we believe that material editing by histogram manipulation suggests a clue to this complex computation. The histogram-matching method, as shown in Fig 1, successfully changes the material appearance of a surface image, while it seems to have a negligible effect on surface shape. This suggests that the image properties changed by the histogram transformation affect material processing, while those unchanged by the histogram transformation affect shape processing. In what follows, we will show which components in the image are changed and unchanged by histogram manipulation and then consider the effects of each component on the perception of material, shape and reflectance change. The analysis will lead us to a computational strategy the visual system may follow to simultaneously and nearly independently estimate material, shape and reflectance change from a single image of an object.

To anticipate the conclusion, we here propose that the human visual system may use orthogonal features about image intensity gradient to estimate material and shape: the intensity gradient magnitude for material perception, and the intensity gradient order for shape perception. We also suggest that the intensity order structure provides the critical information for discrimination of highlights from albedo changes [13–17]. The intensity gradient magnitude is related to the intensity histogram statistics, which some have suggested are related to material perception [8], while the intensity gradient order is related to the isophote and orientation flow that have been suggested to be important for robust shape estimations [19–27]. Combining thoughtful insights originating from past theories with new image analyses and psychophysical experiments, we attempt to comprehensively understand how the human visual system simultaneously estimates many interdependent object properties from a single picture.

## Results

### Image constraints for material changes

To explore image constraints for discriminating material changes from other property changes, we focused on a histogram-transformation method that has been widely used to edit the material appearance of a surface image [1,3,6]. In this method, to adjust the luminance histogram of an original image to that of a reference image, each histogram of the original and reference images is converted into a cumulative histogram (Fig 1(D) and 1(E)). Then, the bin values of the original histogram are transformed into those of the reference histogram so that each cumulative value of the original histogram is matched to that of the reference one. Consequently, histogram matching does not change the intensity order of the image. When the pixel intensity of the output image is plotted as a function of that of the original image (Fig 1F), the tone-remapping function monotonically increases, or at least does not decrease. Similar features are observed in general tone mapping techniques [5]. These observations suggest that retaining the intensity order of the original image may be the key feature for editing material while keeping other physical properties constant.

**Fig 2:**
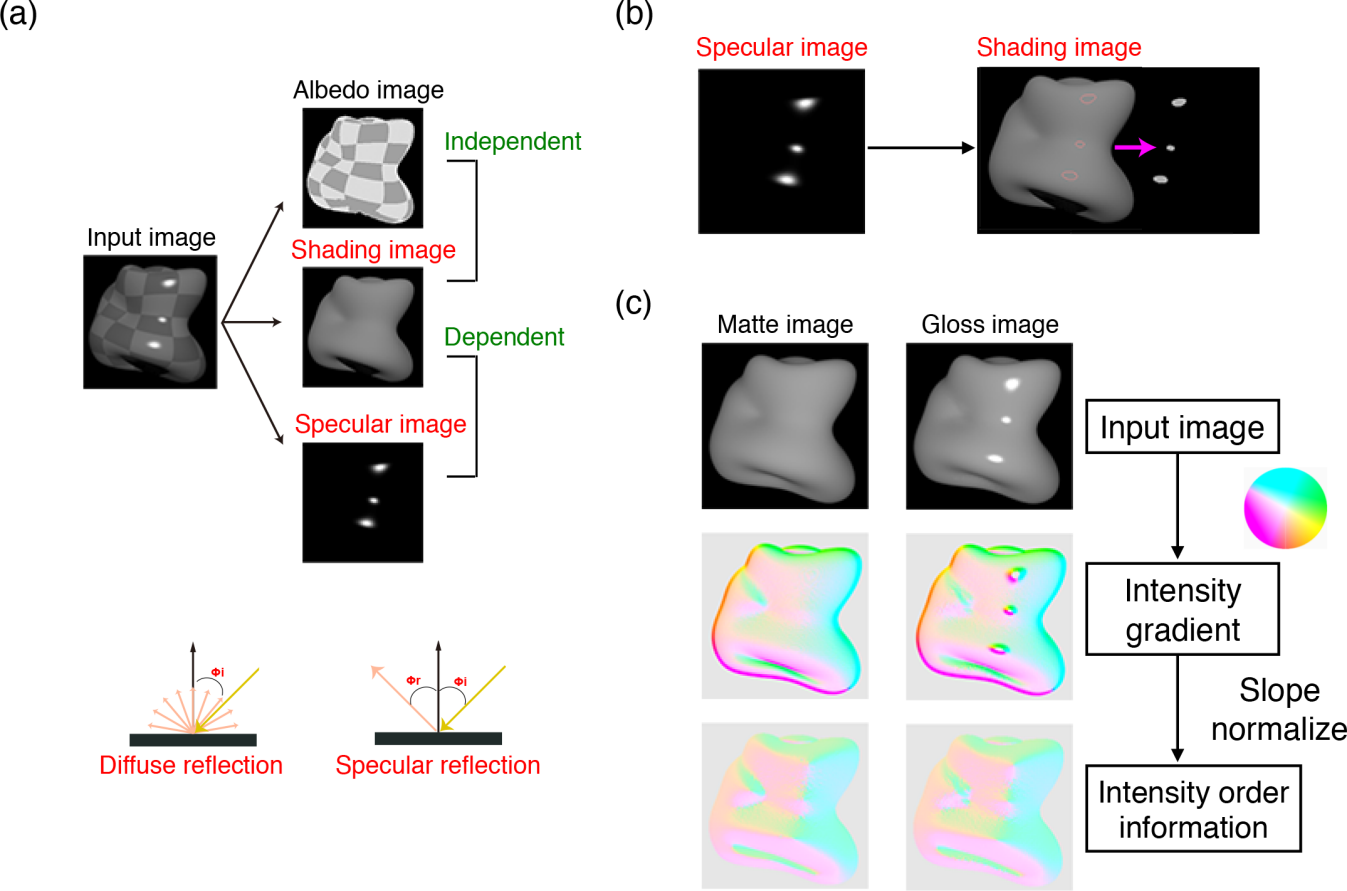
(a) The image is decomposed into its intrinsic components: albedo, shading, and specular images. Whereas the reflectance image is independent of the shading image, the specular image is dependent on the shading image. This is because the shading intensity is a function of the incident angle of light, while the specular intensity is a function of the incident and viewing angles of the light. (b) The intensities of the shading image within the highlight regions tend to be uniform because both specular and shading intensity depend on the incident angle of light. (c) Intensity gradient maps and the direction maps of the intensity gradient obtained from input images. The magnitude and direction of the vector are indicated as the hue and saturation of a color map, respectively.

When we consider the image generation processing of an object image, there are good reasons to believe that retaining the intensity order information is critical for material editing. A (monochromatic) surface image can be decomposed into albedo, shading, and specular images (Fig 2A). The albedo image of a surface indicates how much illumination is diffusely reflected at each surface point. It is irrelevant to the surface normal and thus independent of the shading and specular images. The shading image of a surface is the interaction map of the surface normal and the illumination. With diffuse Lambertian shading, the shading intensity is a function of the incident angle of light. The specular component is the direct mirror-like reflection of the incident light. The specular intensity of a surface is a function of the incident and viewing angles of light.

Since both specular and shading intensities depend on the incident angle of light, the specular image is dependent on the shading image. Under a collimated illumination, the surface normal directions of highlight regions are nearly uniform, and the intensity of the matte component behind the highlight regions is also nearly uniform (Fig 2B). If the highlight regions are uniformly painted with (or replaced by) the hidden matte intensity, the image becomes something akin to a matte surface image. Although the hidden matte intensity is not known, the specular highlights tend to be produced near the highest intensity of the diffuse shading, but the position could shift slightly depending upon the difference between the incident angle and viewing angle [24]. Because of these constraints, adding specular gloss on a matte image, unlike adding an albedo change, has relatively little effect on the intensity order of the image.

If the luminance order structure is the same, so is the isophote structure (an isophote is a contour of equal intensity in an image). The direction of the luminance gradient is orthogonal to the isophote. Hence, if the intensity order information of an image is kept constant, the direction of the intensity gradient is too. Fig 2C shows how adding gloss affects the intensity gradient structures. To make an intensity gradient map, we computed the horizontal and vertical derivatives of the intensity distribution, and then converted them to the polar coordinate. In Fig 2C, the magnitude and direction of the intensity gradient vector are indicated by the hue and saturation of a color map, respectively. The intensity gradient map shows that highlight regions have larger gradient magnitudes, which implies that adding specular highlights drastically changes the gradient magnitudes. However, when the gradient magnitudes are normalized and only the directional information of intensity gradients is preserved, the map of the gloss image is similar to that of the matte image. The results suggest that adding gloss to a matte image has a negligible effect on the direction map of the intensity gradient.

### Image analysis

**Fig 3:**
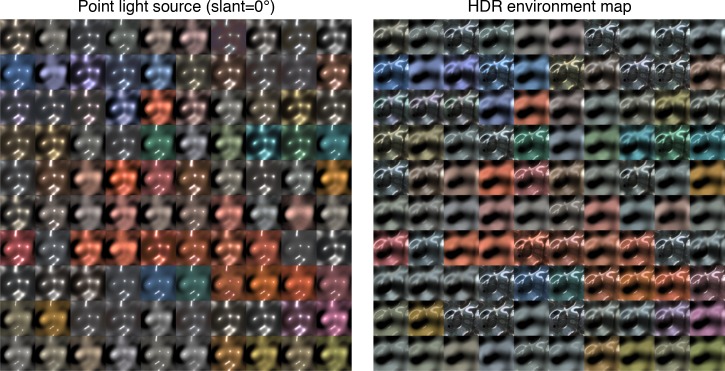
Stimulus examples used in the image analysis. The bumpy surfaces were rendered using the BRDFs in the MERL database (100 BRDFs). The left panel shows the images rendered under a point light source with a slant of 0°, and the right panel shows the images rendered under an HDR environment map. Each panel includes 100 images rendered with different BRDFs. The same geometry is used for the rendered images in the left and right panels. From each rendered image, the direction and the magnitude maps of the intensity gradient were computed.

**Fig 4:**
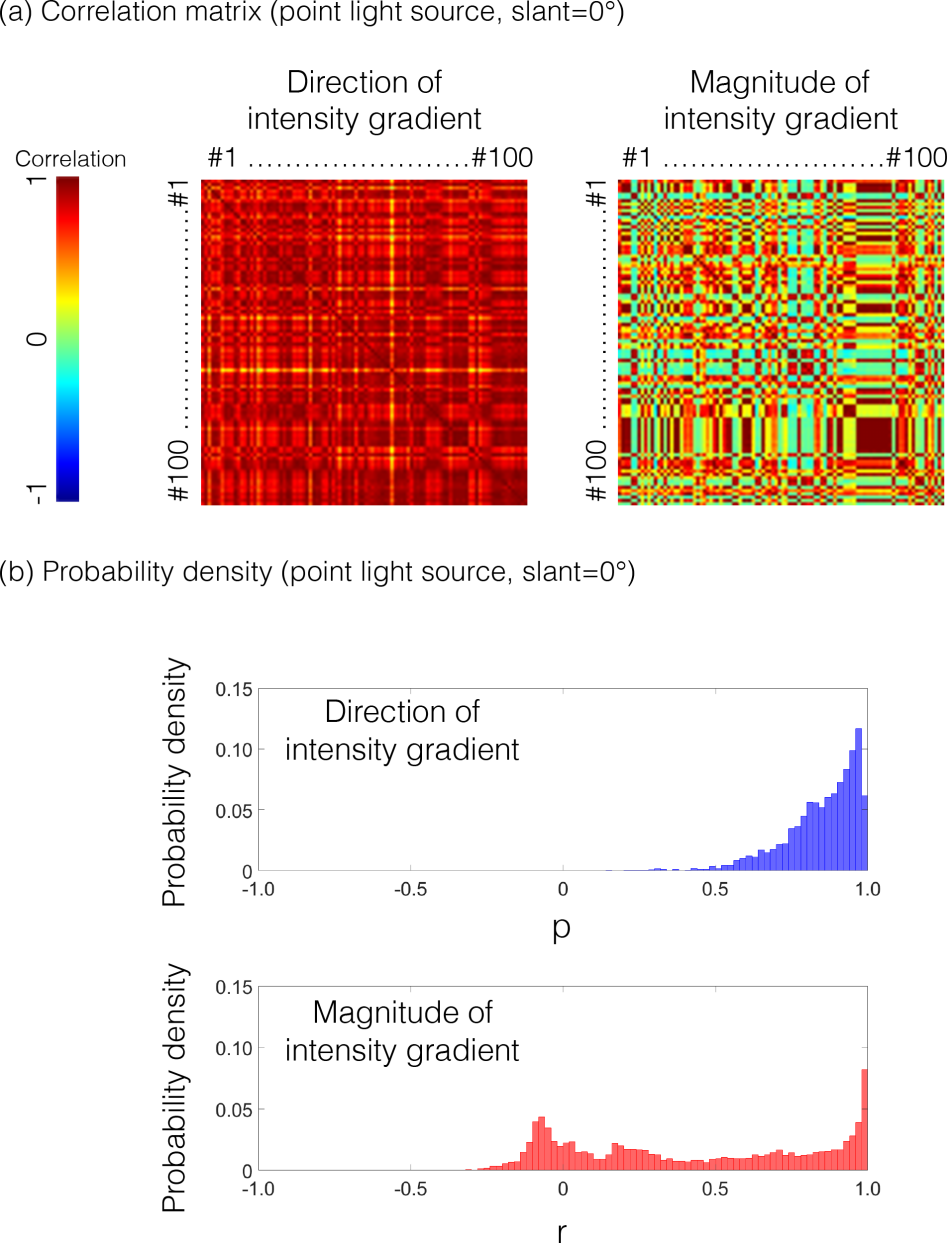
Probably density distributions of correlation coefficients between all pairs of BRDFs. (a) The left and right panels show the correlation coefficients on the direction and the magnitude of the intensity gradient of the rendered image, respectively. For the direction condition, the circular correlation was used [29, 30]. Each cell of the panels indicates the correlation coefficient between the BRDFs of rows and columns. The numbers from #1 to #100 indicate the index of MERL BRDFs. (b) The probability density distribution of the correlation coefficients. The top panel indicates the correlation of the direction of the intensity gradient. The bottom panel indicates the correlation of the magnitude of the intensity gradient. When the point light source lit the objects from the viewing direction, the direction of the intensity gradient consistently shows quite a high correlation, whereas the magnitude of the intensity gradient shows correlations that are relatively low and highly variable depending on the comparison pair.

To test the generality of the observations, we analyzed material images rendered using the MERL BRDF (Bidirectional Reflectance Distribution Function) database [28], which is a set of measured BRDFs of 100 materials, including rubber, plastic, metal, and fabric. There were four illumination conditions: three single point light sources (slant = 0, 20, and 40 degrees) and one HDR (High Dynamic Range) environment map (Fig 3). Fig 4A shows the results of the analysis. Each cell of the panels indicates the correlation coefficient of the magnitude or the direction map of the intensity gradient between the images rendered with the BRDFs of the row and the column. When the point light source lit the objects from the viewing direction, the direction of the intensity gradient consistently showed quite a high correlation (Fig 4(B), upper), whereas the magnitude of the intensity gradient showed correlations that are relatively low and highly variable depending on the comparison pair (Fig 4(B), bottom). These findings can be confirmed from the probability density distribution of the correlation coefficients in Fig 4(B).

**Fig 5:**
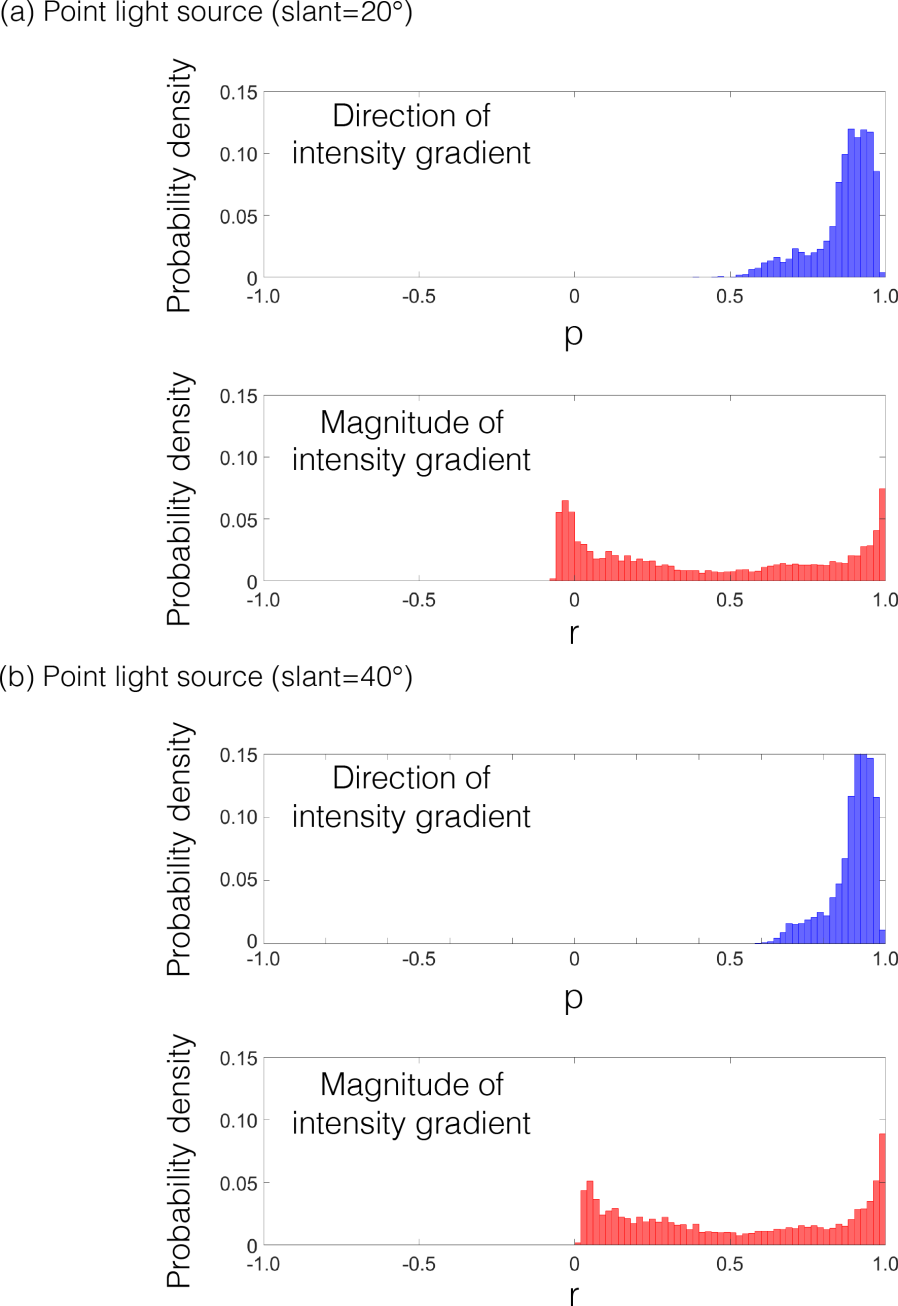
Probability density distributions under the lighting conditions of (a) 20° and (b) 40°. The displacements of the lighting direction do not significantly change the pattern of the correlation distributions.

When the incident angle of the light deviates from the viewing angle, the position of specular highlights tends to shift from the position of the highest intensity of the diffuse shading [24]. However, the displacements of the lighting direction do not significantly change the pattern of results (Fig 5(A) and 5(B)). The analysis of the surface images rendered under point light sources suggests that material changes (with no changes in shape and illumination) have little effect on the direction of the intensity gradient.

**Fig 6:**
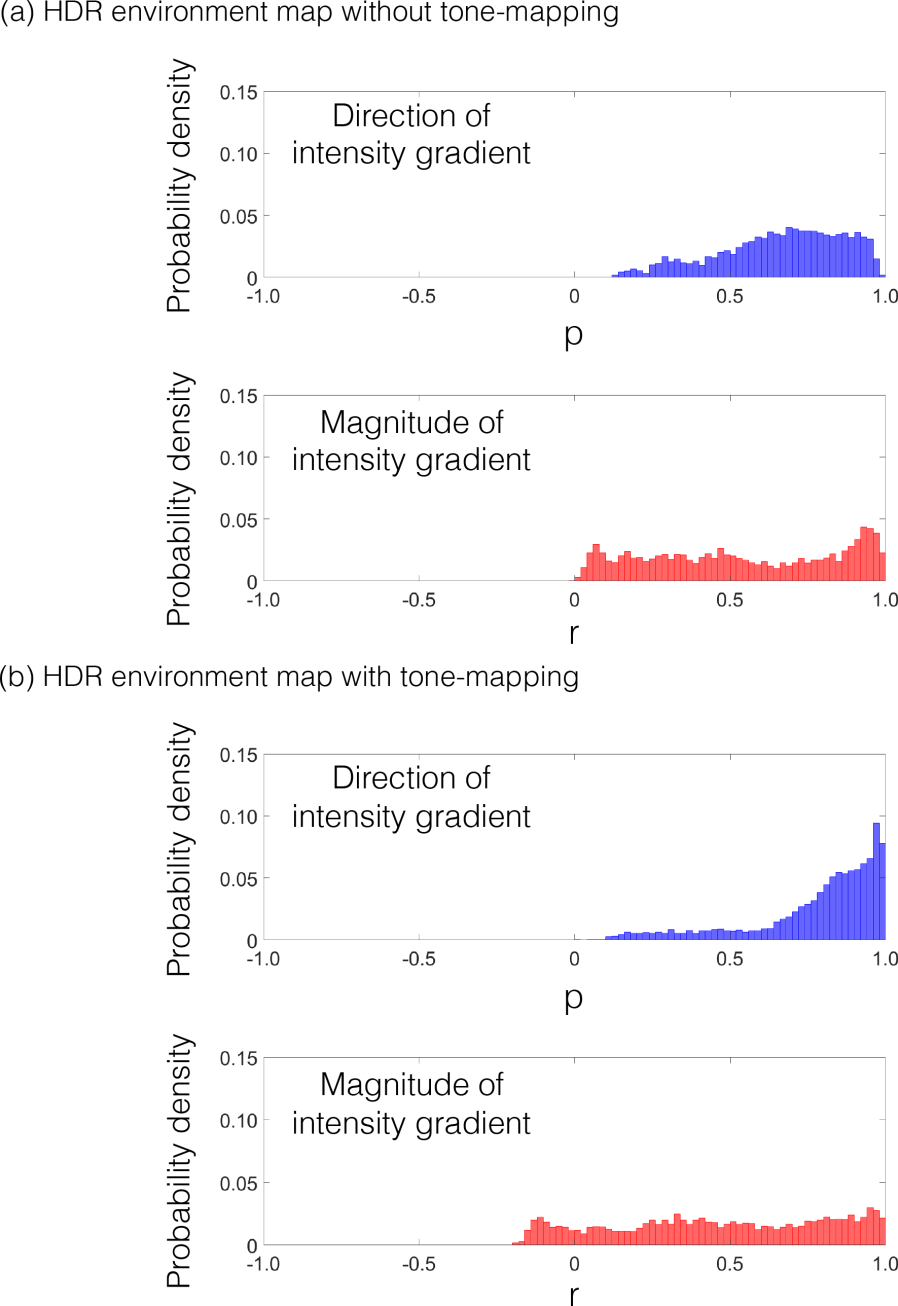
(a) Probability density distributions in the HDR environment map. The correlation in the direction of the intensity gradient is reduced for some material combinations. (b) The correlation of the image in the HDR environment map was calculated after applying the compressive tone-mapping of Eq (1) to the surface image. As a consequence, the correlation in the direction of the intensity gradient is markedly improved.

Under the HDR illumination environment, the correlation in the direction of the intensity gradient is reduced for some material combinations (Fig 6(A)). This is because the direction of the intensity gradient is disturbed by the spatially complex illumination that produces spatially non-uniform mirror reflections, especially when the BRDF has low specular roughness. Since we computed the intensity gradient on a small scale (the kernel size was 5 x 5 pix for an image size of 256 x 256 pix), the fine structures of a mirror reflection of the environment affect the direction of the intensity gradient. However, a simple tone operation can reduce the effect of a mirror reflection of the environment. While tone remappings normally change the intensity histogram without changing the intensity order, strong compressive tone remappings in which the output intensity levels off beyond a certain input intensity can remove the intensity gradients in the high intensity range. Since the mirror reflection generally has higher intensities than those of the shading pattern, a strong compressive tone mapping can eliminate the variation caused by the spatially non-uniform mirror reflection. In one analysis (Fig 6(B)), when the magnitude became smaller than a very small threshold value, we excluded the gradient values from computation of gradient directions. The analysis showed that the correlation in the direction of the intensity gradient is markedly improved.

**Fig 7:**
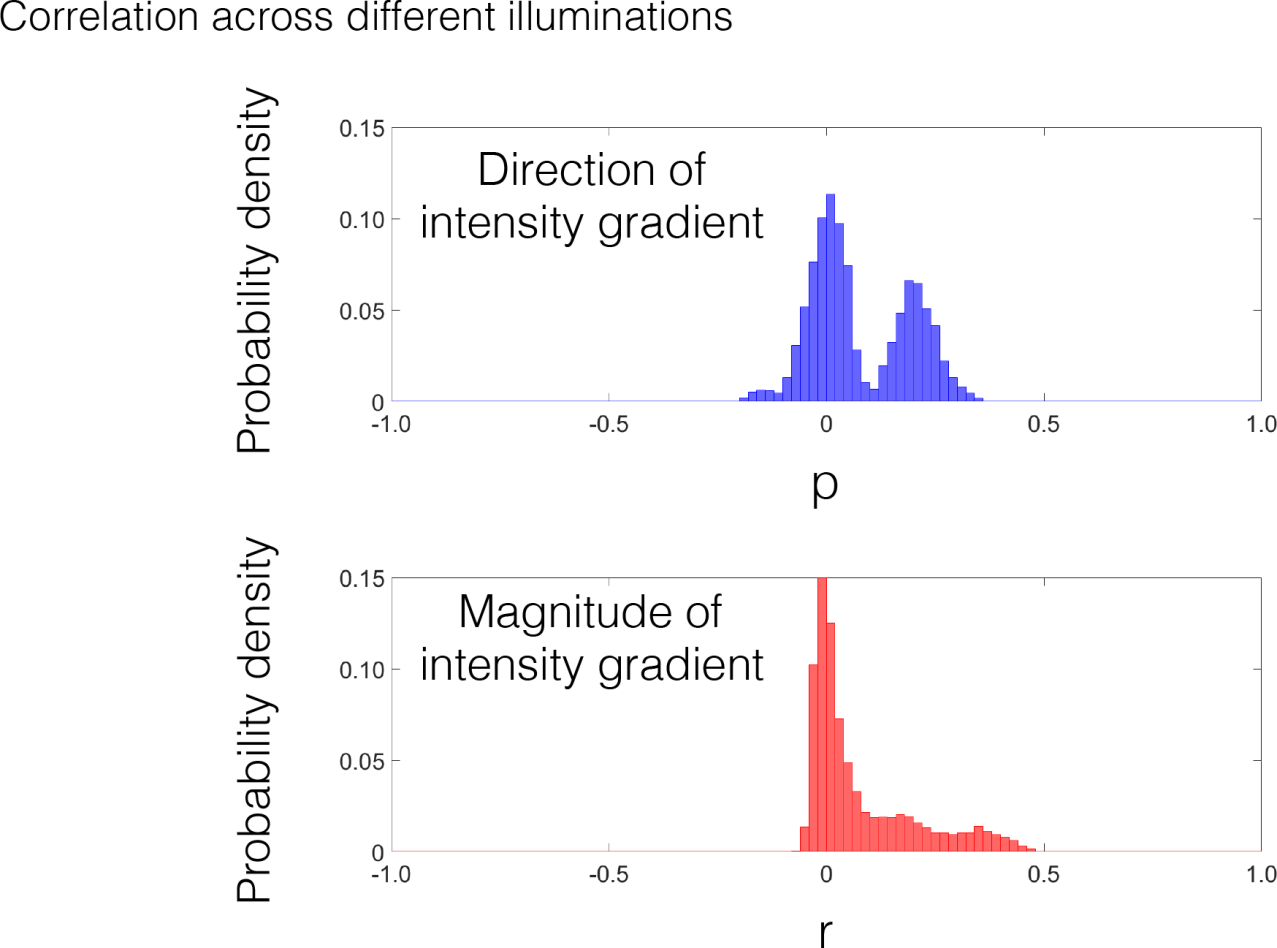
Probability density distributions of the correlation between the lighting conditions of 0° and 40°. The correlations in the direction of the intensity gradient are much lower compared with those under the identical illumination environment condition (Figs 4–6).

In addition, it should be noted that the strong correlations in the direction of the intensity gradient across different materials can be obtained only under similar illumination conditions. When we compute the correlation across different illuminations, e.g., between the lighting conditions of 0° and 40°, the direction information of the intensity gradient is markedly different (Fig 7).

The present analysis suggests that the material change of a surface strongly modulates the magnitude of the intensity gradient but does not unduly disrupt the intensity order or the direction of the intensity gradient. This explains why the histogram-matching method, which affects the magnitude of the intensity gradient while preserving intensity order, effectively changes the material appearance.

At the same time, our analysis suggests that the intensity order of an object image contains rich information about the surface shape and reflectance pattern. In the context of computer vision, intensity order information is widely utilized as a feature descriptor [31–34]. For instance, Dalal and Triggs [31] utilized the local histograms of image gradient orientation (called histograms of oriented gradient, or HOG). They showed that the descriptor is robust against environmental changes. In addition, shape-from-shading studies suggest that the intensity order information or the directional information of the intensity gradient is useful for shape estimation [19–27].

**Fig 8:**
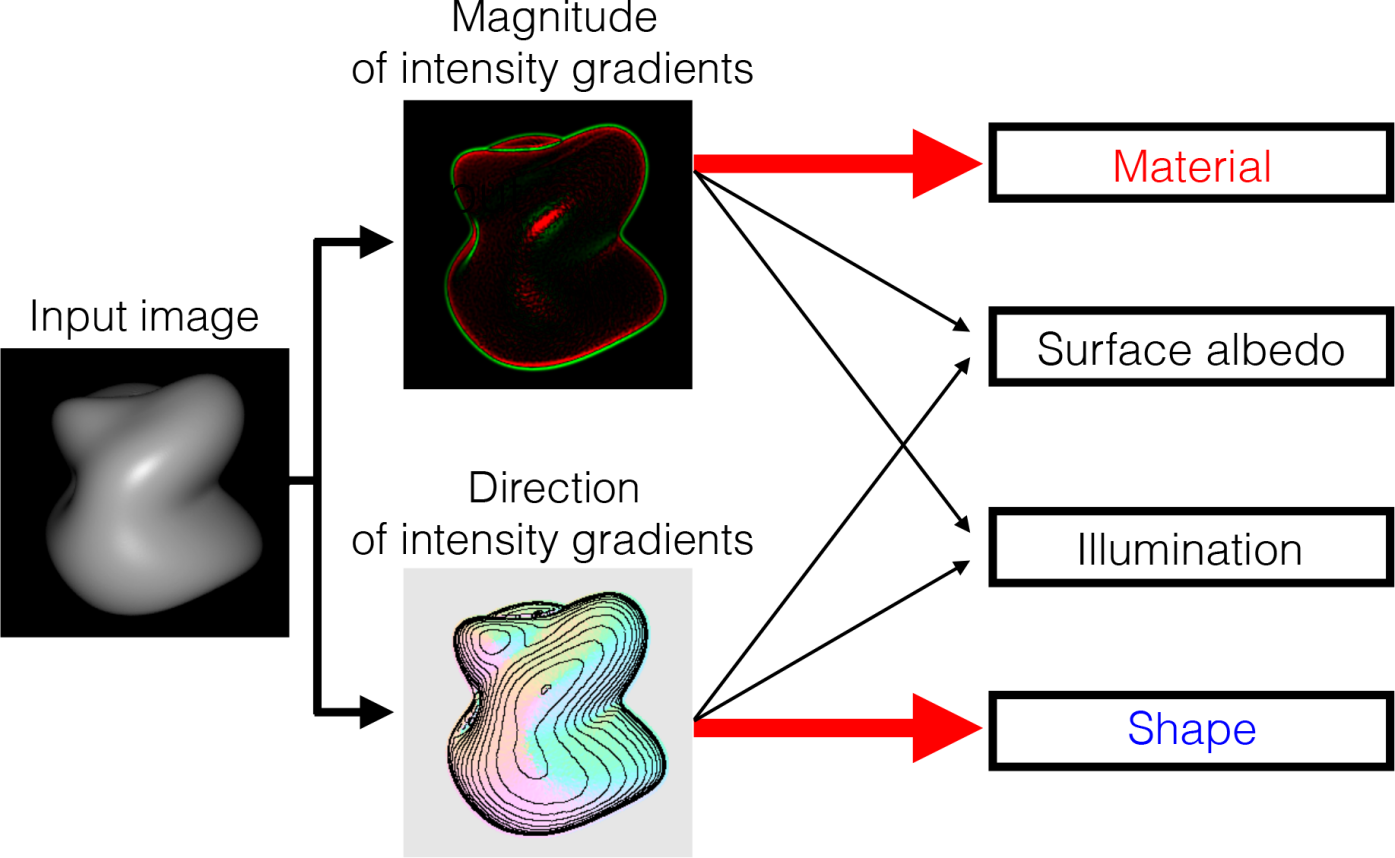
A hypothetical processing scheme that the human visual system may use for simultaneous estimation of a variety of surface properties. We hypothesize that human shape processing is more sensitive to image features given by the intensity order information than those conveyed by the gradient magnitude information, while the magnitude information is dominantly used for material processing.

Fig 8 shows a hypothetical processing scheme that the human visual system may use for simultaneous estimation of a variety of surface properties. The critical idea is that an input surface image is analyzed in two ways. One focuses on the information about the order of intensity. It could be in the form of isophote, gradient direction map, or orientation map. Shape processing mainly relies on this intensity order information. The other image analysis focuses on the information about the magnitude of the intensity gradient. Material processing mainly relies on this gradient magnitude information. To be precise, the important information for material estimation is likely to be the intensity gradient relative to the surface orientation change [18], but we assume this is computed in subsequent stages. The estimation of the remaining properties, i.e., surface albedo and illumination, relies both on the intensity order and gradient information, along with the absolute intensity level. According to this hypothesis, one can tell whether a bright spot is produced by an albedo change or by a specular highlight by checking how it affects the intensity order information.

While previous studies have suggested that luminance histogram manipulation is an effective way to change material appearance [8], as well as pointing out the importance of orientation field or isophote map in shape perception [19–27], to our knowledge, one potential implication of these findings has not been recognized. That is, material perception and shape perception may be based on separate, independent, orthogonal features of the object image, and this is why the visual system can simultaneously estimate material and shape. Although visual estimation of the material and shape appears to include a hard chicken-and-egg problem (material estimation requires shape information, while shape estimation requires material information), the brain may be able to solve it by computing the two attributes, at least initially, based on the independently measurable image features.

Although our hypothesis includes an explanation as to how the visual system robustly estimates the shape for some materials, it does not cover every kind of material. This is because our basic intuition came from a critical observation that luminance histogram matching affects apparent material, but not shape. While luminance histogram matching realized by monotonic luminance re-mapping can produce a wide range of matte and glossy objects, it cannot easily make mirrored objects with a perfectly specular reflectance. Hence, we do not have a strong theoretical basis to assume that our theory is applicable to mirrored objects. Textured objects and line drawings are also outside our scope. Compared to the “orientation field” theory proposed by [23–25], we consider a more specific problem of monocular shape perception (see Discussion for details).

In the five psychophysical experiments reported below, we empirically tested our hypothesis. The first three experiments measured the apparent shape (surface orientation) of object images to see whether it is affected by the intensity order information but not by the intensity gradient magnitude information. Experiment 1 changed the intensity distribution by means of histogram matching that preserved the intensity order information. Experiment 2 changed the intensity distribution by means of non-linear intensity remapping that disrupted the intensity order information under some conditions. Experiment 3 disrupted the intensity order information more naturally by using velvet-like surface reflectance. The last two experiments examined apparent surface gloss and reflectance uniformity to ascertain whether they are affected by the intensity gradient magnitude information but not by the intensity order information. By using objects with veridical and inconsistent highlights, we considered how the two types of intensity information are used to discriminate material features from reflectance changes. Experiment 4 changed the intensity distribution by means of histogram matching, while Experiment 5 changed it by means of compressive remapping.

### Perception of shapes

#### Experiment 1

**Fig 9:**
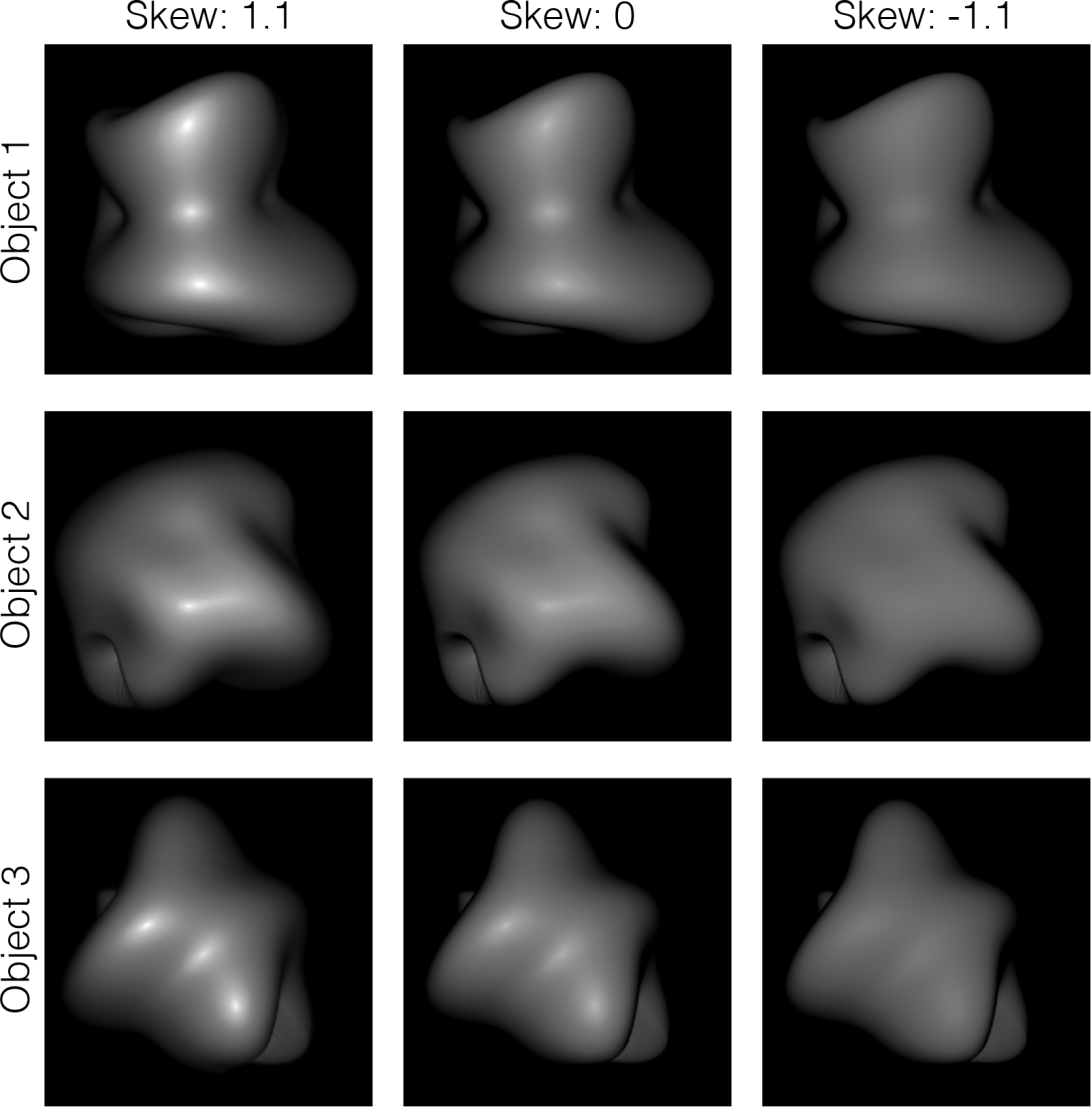
Stimuli used in Experiment 1. The skewness of the intensity histogram of each object image with highlights was modulated using a standard histogram matching method.

The first experiment examined how modulating the intensity distribution of the object images affects its shape appearance. We used a histogram matching method, which affects the intensity gradient magnitude information but not the intensity order information. The object had diffuse reflection or specular reflection producing veridical highlights. The skewness of the intensity histogram of these object images was modulated by using the histogram matching method (Fig 9). We measured the perceived shape using a “gauge probe” task [35]. For comparison, we also measured the perceived glossiness in a rating task.

#### Glossiness judgment

**Fig 10:**
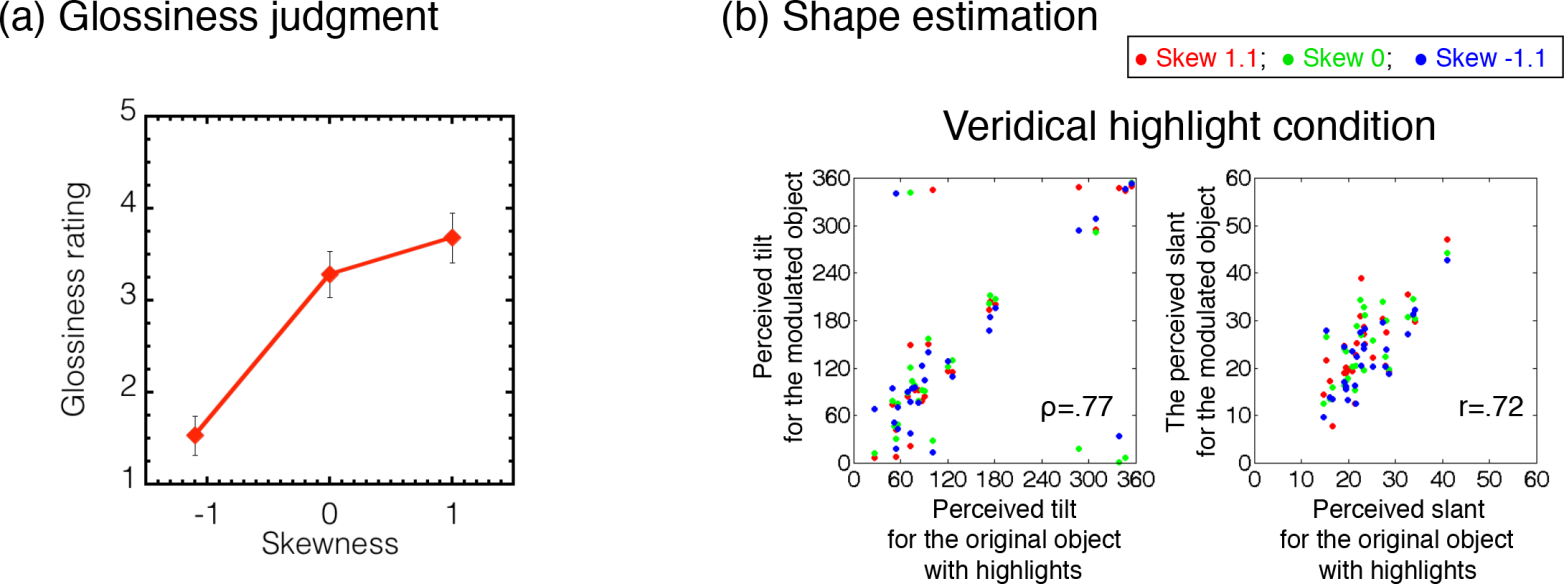
Results of Experiment 1. (a) Rating results for the glossiness judgment plotted as a function of the skewness of the intensity histogram. Error bars indicate ± 1 SEM across observers. (b) The perceived tilt (left) or slant (right) for the histogram-modulated object plotted as a function of those for each original object. Different symbols indicate different skew parameters as shown in the legend.

Fig 10(A) shows the results of the glossiness ratings for the object images, plotted as a function of the skewness of the intensity histogram of the object images. Each symbol indicates the rating values averaged across different observers. Error bars denote ± 1 SEM across observers.

Results show that the perceived glossiness changed with the skewness of the object images. Specifically, when the intensity histogram of the object image was negatively skewed, the image did not look glossy at all. In contrast, when the intensity histogram of the object image was positively skewed, the image looked glossy. The effects of histogram skewness on gloss perception are consistent with the findings of Motoyoshi et al. [8].

#### Shape estimation

Fig 10(B) shows the results of the effects of histogram manipulations on surface shape perception by scatter plots between the perceived shapes of the histogram-modulated objects and those of the original objects. For each observer, each gauge position, and each stimulus condition, we averaged the perceived tilt or slant of the gauge probe across trials. The perceived tilt (left) or slant (right) of the histogram-modulated object is plotted against that for the original object in Fig 10(B). In the figure, different color symbols indicate different skew parameters. The results show high correlations between the perceived shapes of the histogram-modulated and original objects, regardless of the histogram skewness. That is, the perceived shape of an object image was barely affected by the intensity histogram as long as the intensity order was not disrupted, in agreement with the idea that human shape processing is sensitive to intensity order information but not sensitive to intensity gradient magnitude information. The results are consistent with the previous finding showing that adding specular highlights to a matte object does not change the perceived shape [36, 37].

#### Experiment 2a

**Fig 11:**
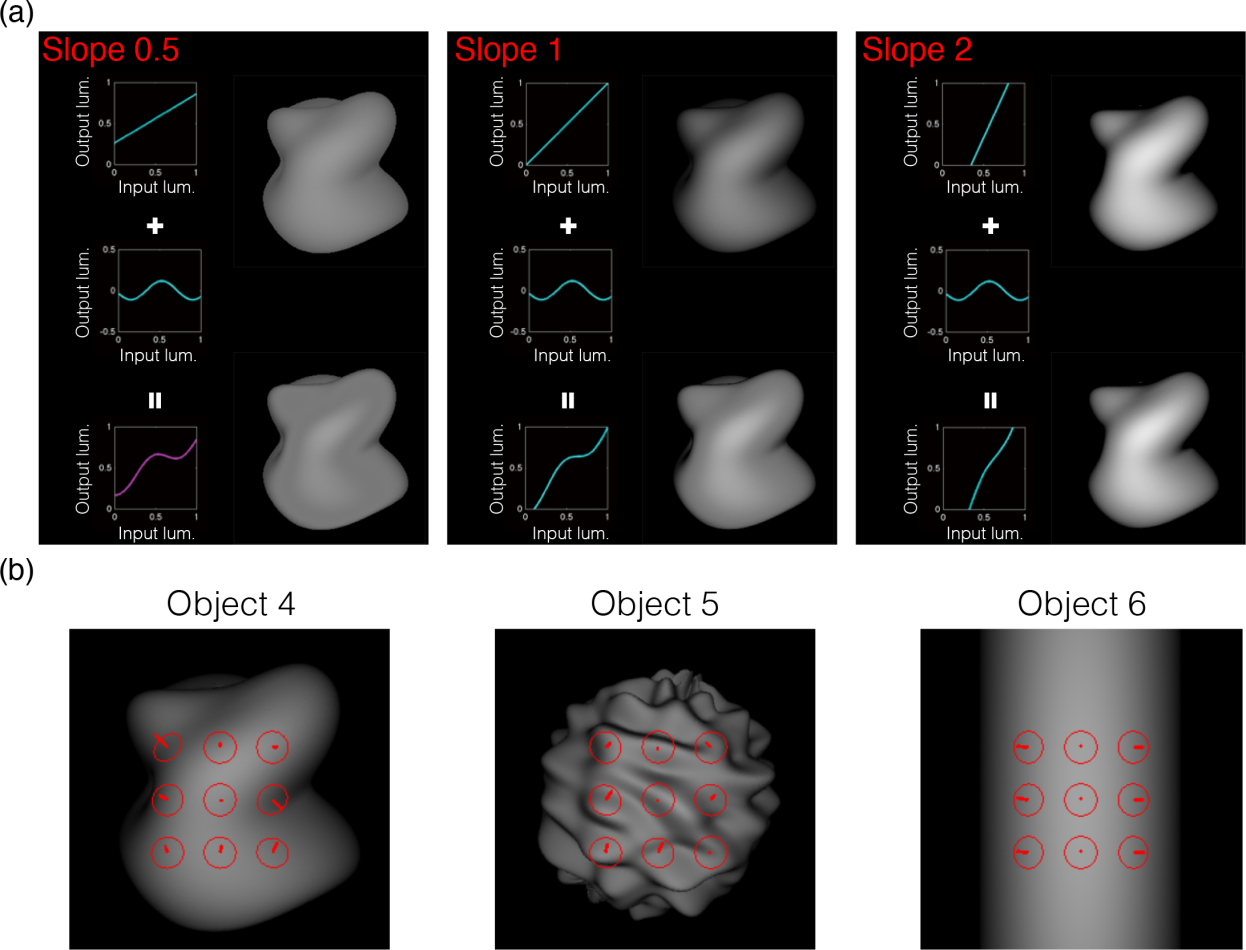
Stimuli used in Experiment 2a. (a) We applied a variety of monotonic and nonmonotonic nonlinear remappings to several object images. When the slope was steep (right), the intensity order of the original image was not disrupted by the modulation regardless of its amplitude. In contrast, when the slope was gentle (left), the intensity order was disrupted by the modulation unless the modulation amplitude was small. When the intensity order of the original image was disrupted (left bottom), the shape of the image was perceived differently from the original one (middle bottom). (b) The three object images used in Experiment 2. The gauge probes on the image show the position measured in the experiment.

Experiment 1 showed that holding intensity order information did not change the perceived shape. In Experiment 2a, we investigated what happens to shape perception when the intensity order of an object image is disrupted (Fig 11). In the experiment, while applying a variety of non-linear remappings on several object images, we estimated the perceived shape of the objects by asking observers to set a gauge probe to match the apparent surface slant/tilt. Specifically, linear tone curves having several slopes (0.5, 1, and 2) were modulated by a sinusoidal modulation of four different amplitudes. When the slope was steep, the intensity order of the original image was not disrupted by the modulation regardless of its amplitude. In contrast, when the slope was gentle, the intensity order was disrupted by the modulation unless the modulation amplitude was small.

**Fig 12:**
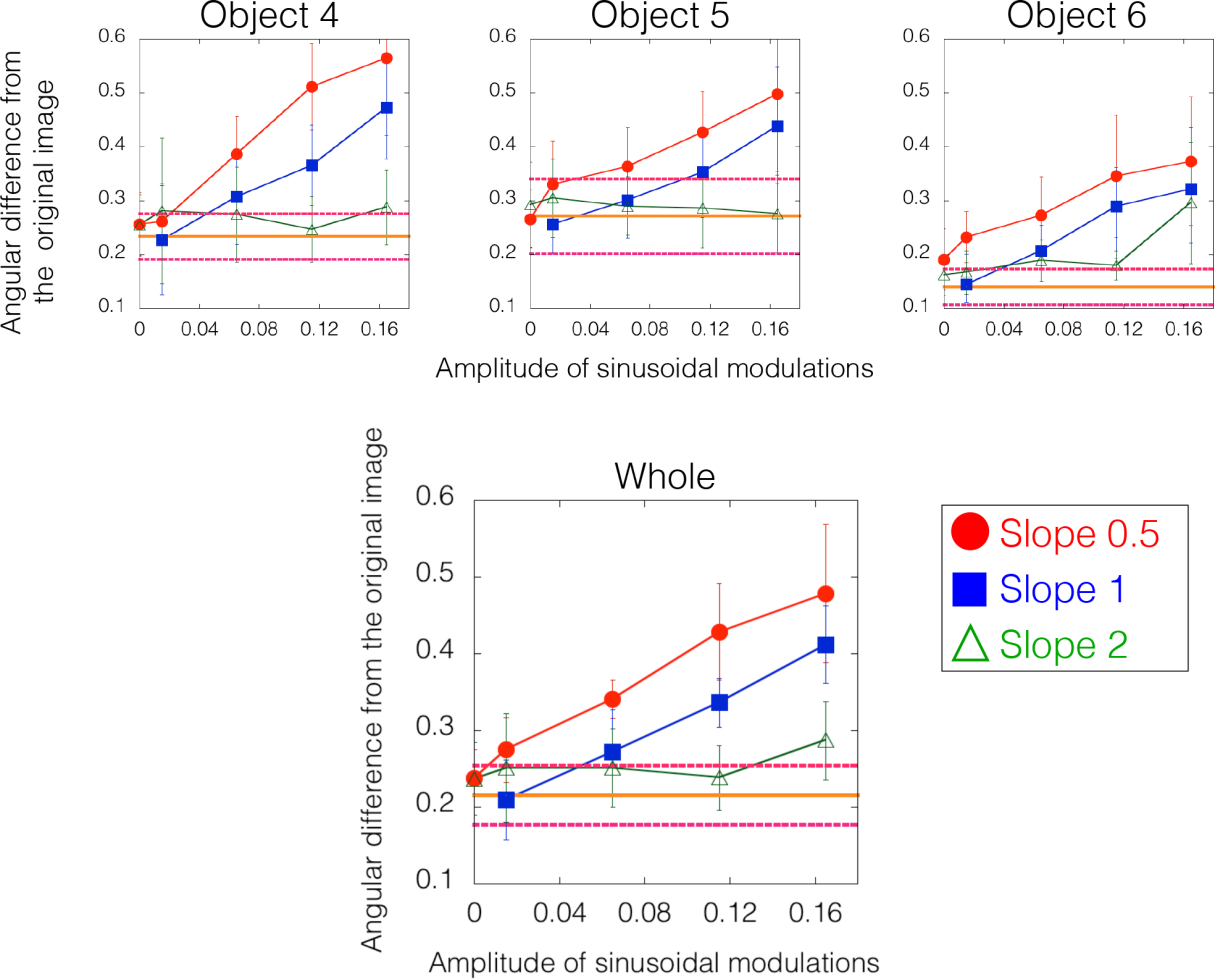
Results of Experiment 2a. The angular difference between the judgments on tone-mapped and original images is plotted as a function of the amplitude of the sinusoidal modulation. Error bars indicate 95% confidence intervals computed using bootstrap estimates. Different symbols indicate different slopes as indicated in the legend. The orange (solid) horizontal line shows the mean angular difference of matched gauges for the same original object measured in different sessions (i.e., the control condition). Magenta (dashed) horizontal lines indicate 95% confidence intervals of the control condition computed using bootstrap estimates. For slope 1, the intensity order of the original image was disrupted when the sinusoidal modulation was 0.115 or 0.165. For slope 0.5, the intensity order was disrupted when the sinusoidal modulation was 0.065, 0.115 or 0.165. For slope 2, the modulations did not change the intensity order. Results show that the large deviations of the perceived shape were obtained when the intensity order of the original image was markedly disrupted.

Fig 12 shows the results of the experiment. The angular differences in the tone-mapped images from the original image are plotted as a function of the amplitude of the sinusoidal modulations. For each stimulus condition, the angular difference was calculated for each gauge probe within each observer, and the nine angular differences were averaged across the gauge probes. Then, the mean angular differences were averaged across observers. Error bars indicate 95% confidence intervals computed using bootstrap estimates. The solid horizontal line shows the angular difference for the same original object measured in different sessions (i.e., the control condition). Magenta (dashed) horizontal lines indicate 95% confidence intervals of the control condition computed using bootstrap estimates.

Results show that the perceived shape of the tone-remapped images was similar to that of the original images unless their intensity order was disrupted. Specifically, when the slope of the linear tone curve was gentle (0.5), the perceived shape of the tone-remapping images was markedly different from that of the original image even when the modulation amplitude was small (i.e., *a*_*1*_ = 0.065). In contrast, when the slope was steep (2), the perceived shape of the tone-remapped images was not affected by the addition of the modulation. The modulation effects were slightly different across the three object images, but the order of the effects was consistent. The findings suggest that the disruption of the intensity order information affects the perceived shape.

#### Experiment 2b

**Fig 13:**
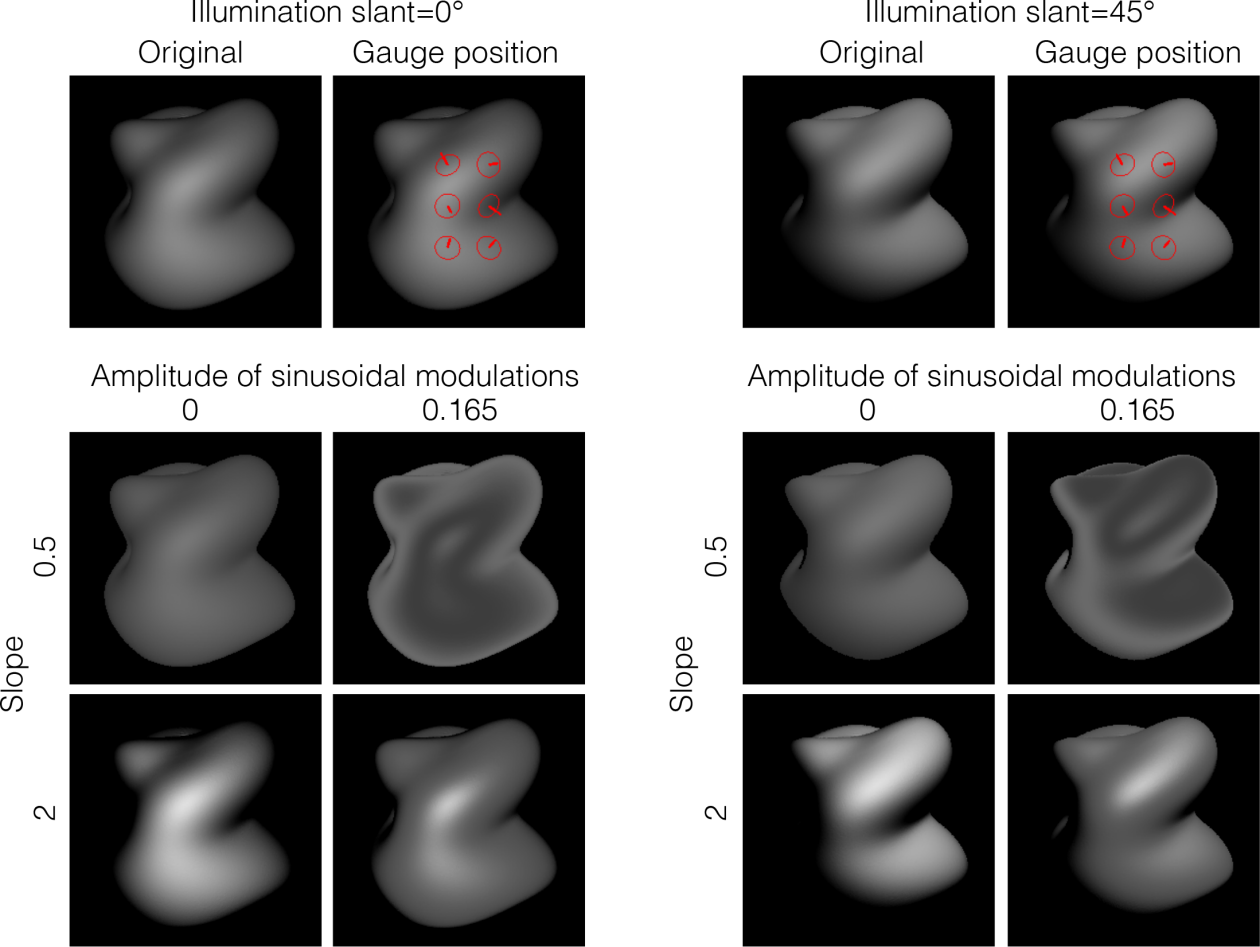
Stimuli used in Experiment 2b. We applied a variety of monotonic and nonmonotonic remappings to the object images under the lighting conditions of slant 0° (left) and slant 45° (right). When the slope of the remapping was steep (i.e., slope = 2), the intensity order of the original image was not disrupted by the amplitude modulation (0.165) as in Experiment 2. In contrast, when the slope was gentle (i.e., slope = 0.5), the intensity order was disrupted by the modulation. Object 4 was used in the experiment. The six gauge probes as shown in the gauge position figures were used.

In Experiments 1 and 2a we used the object images under a point light source placed in the same direction as the viewing direction (i.e., illumination slant = 0°). We additionally conducted Experiment 2b to see whether the effect of the intensity order is affected by the illumination direction. We applied a variety of non-linear remappings, as in Experiment 2a, on an object image (Object 4) rendered under a point light source placed in the front direction (illumination slant = 0°) or in the upper-right direction to the object (illumination slant = 45°) (Fig 13). For both illumination conditions, the intensity order of the original image was disrupted by the amplitude modulation when the slope of the remapping was gentle (i.e., slope = 0.5), but not when the slope was steep (i.e., slope = 2). As before, we asked observers to set a gauge probe to match the apparent surface slant/tilt.

**Fig 14:**
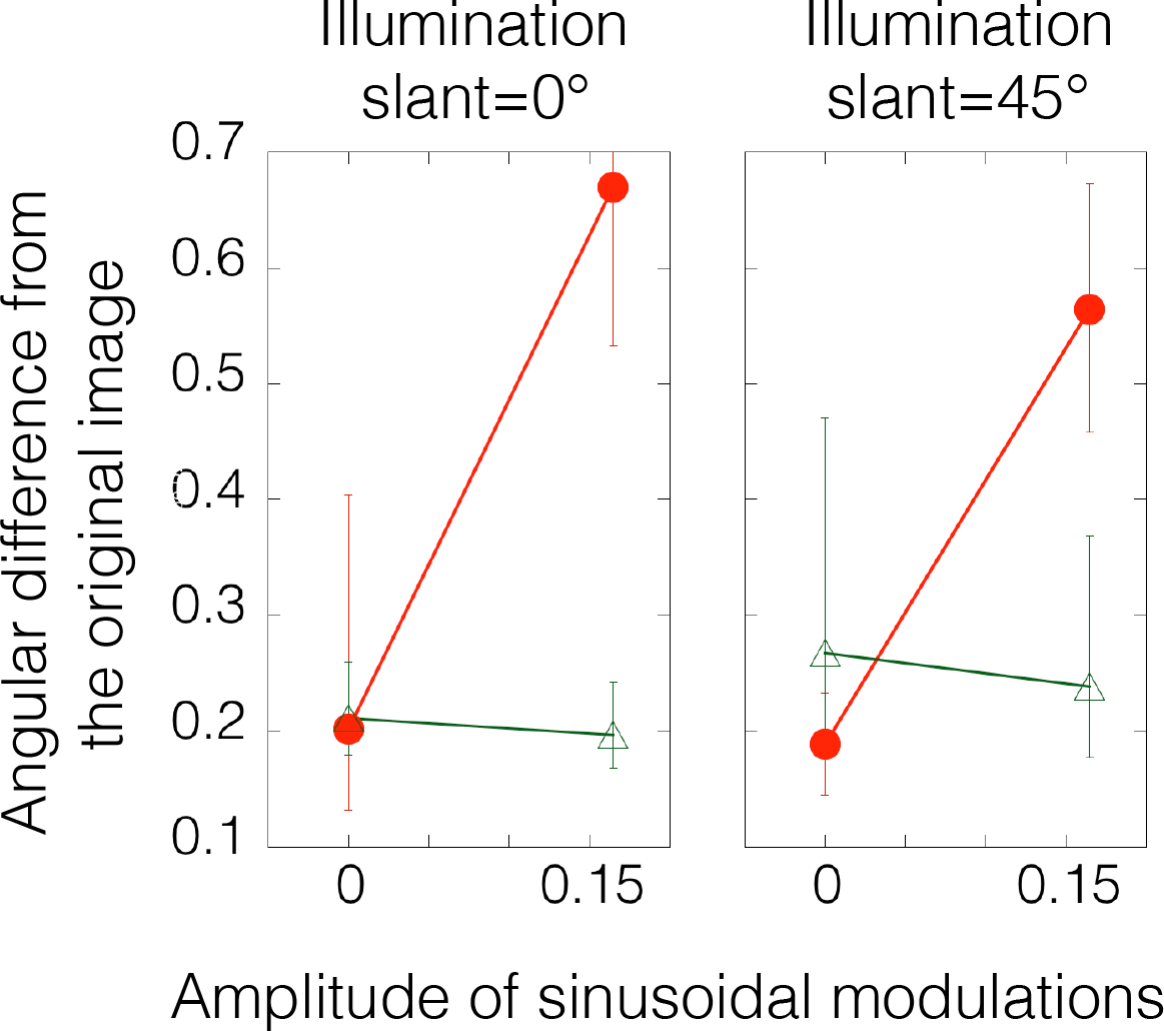
Results of Experiment 2b. The angular difference between the judgments on tone-mapped and original images is plotted as a function of the amplitude of the sinusoidal modulation. Error bars indicate 95% confidence intervals computed using bootstrap estimates. Different symbols indicate different slopes as indicated in the legend of Fig 12. For both illumination conditions, results show that large deviations in the perceived shape were obtained when amplitude modulation was added to the remapping with a slope 0.5, i.e., when the intensity order of the original image was disrupted.

Fig 14 shows the results of the experiment. The angular differences in the tone-mapped images from the original image are plotted as a function of the amplitude of the sinusoidal modulations. The way in which each angular difference was averaged was the same as in Experiment 2a (Fig 12). Results were similar to those of Experiment 2a for both illumination conditions. Specifically, when the slope of the linear tone curve was gentle (0.5), the perceived shape of the tone-remapping images was markedly different from that of the original image due to the addition of the amplitude modulation. In contrast, when the slope was steep (2), the perceived shape of the tone-remapped images was not affected by the addition of the modulation. Again, the findings are consistent with the notion that the intensity order information is critical to how the shape is perceived. In addition, it is noteworthy that the mean angular difference across observers between the judgments of original images with different illuminations (0° and 45°) was 0.25 (95% confidence interval: 0.21–0.31). This small difference in value is comparable to those under the order-retained conditions, although the intensity order information across different illuminations is markedly different. The finding suggests that the visual system is adept at discounting the influence of the illumination field in recovery of the perceived shape from the intensity order information.

#### Experiment 3

**Fig 15:**
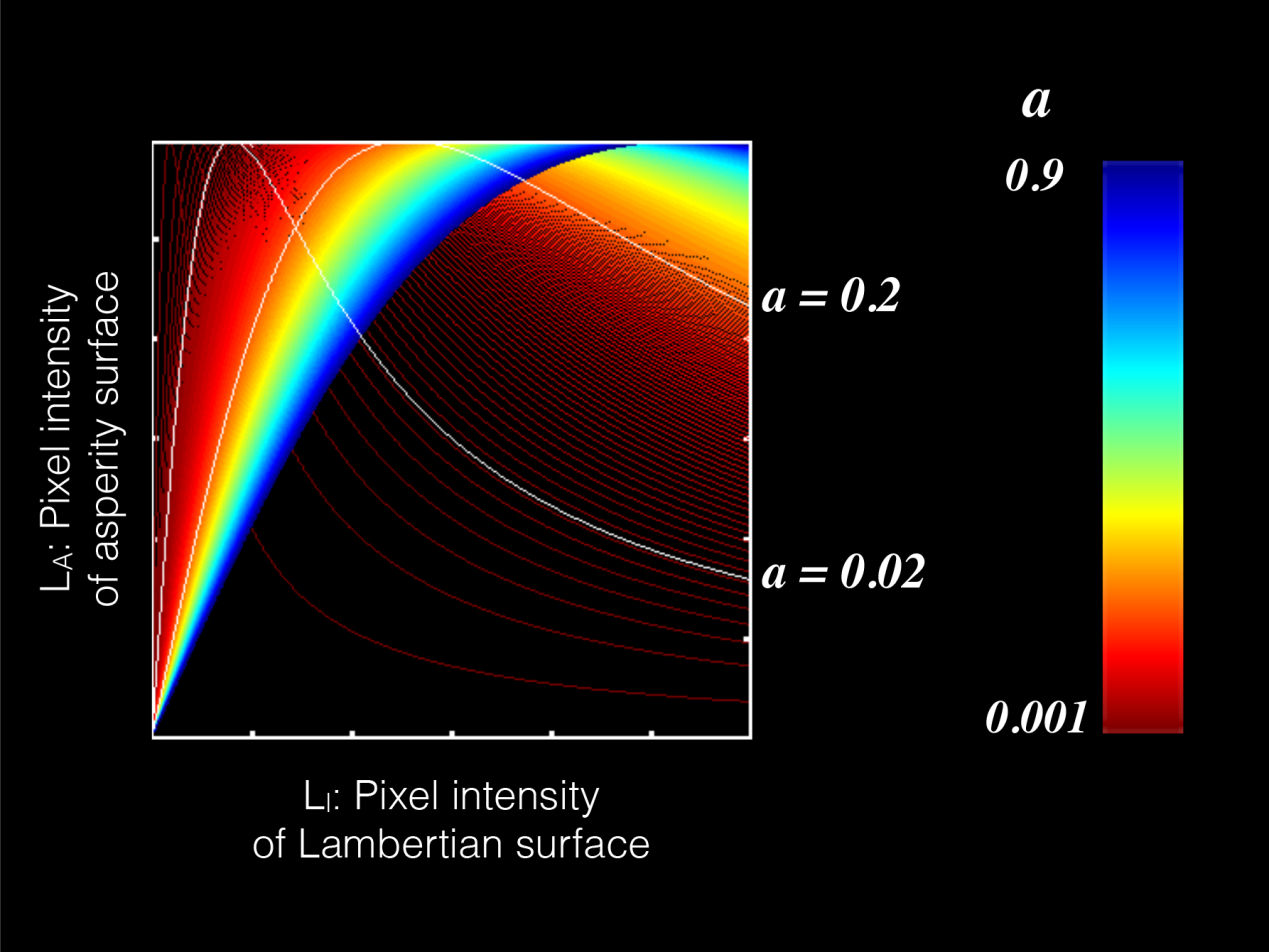
Relation between pixel intensities of Lambertian and asperity surfaces. We assume that the incident and reflected angles of the illumination are the same. Each plot of the L_A_ is scaled from 0 to the max intensity of the L_l_. The intensity order of each plot changes with parameter *a*. For instance, when parameter *a* is 0.2, the intensity order is preserved in the lower range of Lambertian pixel intensity, while it is reversed in the higher range. When *a* is 0.02, the intensity order is reversed across most of the intensity range.

**Fig 16:**
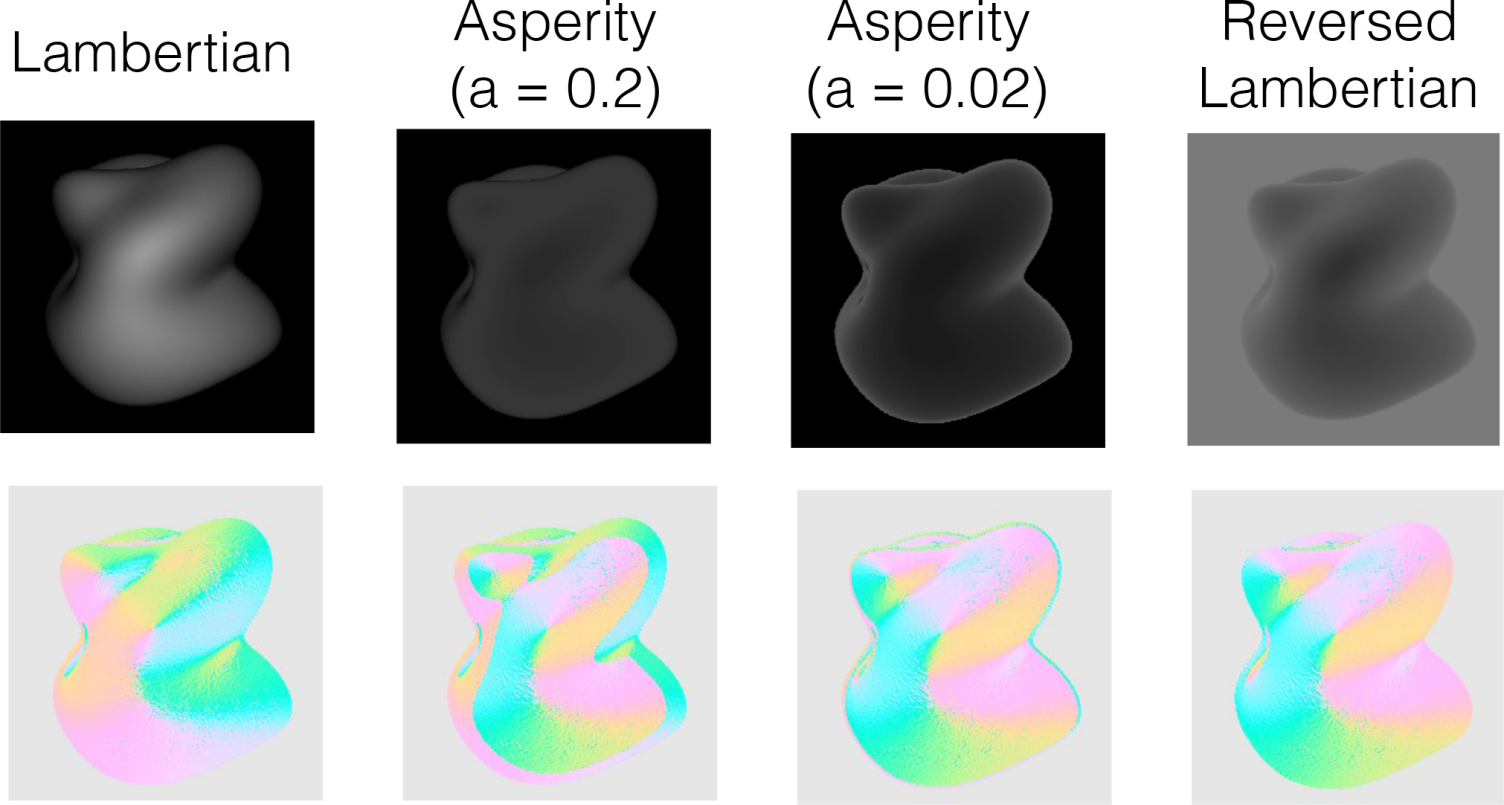
Stimulus examples used in Experiment 4. Upper images indicate the experimental stimuli with different BRDFs as in the legend. Lower images indicate the directional map of each image.

While the Experiments 2a & 2b showed that disrupting the order of the intensity gradient altered apparent shape, our stimulus manipulation was somewhat unnatural. If a similar disruption of intensity order is produced naturally, different results might be obtained. Although most BRDFs in the MERL database do not affect the intensity order information (Fig 4), some materials do; velvet is one example. To elucidate the effect of a change in material that alters intensity order information, we compared the perceived shape between velvet and Lambertian materials. As the reflectance model of velvet materials, we used the asperity scattering BRDF model [38, 39]. For the asperity model, two values of the asperity parameter *a* (*a* = 0.2 or 0.02) were used (Figs 15 and 16). When *a* is moderately small (0.2), the intensity order is preserved in the lower range of Lambertian pixel intensity, while it is reversed in the higher range. When *a* is even smaller (0.02), the intensity order is reversed across most of the intensity range. In addition to a Lambertian object and two asperity objects, we used an object the intensity of which was completely reversed from the Lambertian object (Fig 16). In the experiment, we measured the perceived shape in a gauge probe task.

**Fig 17:**
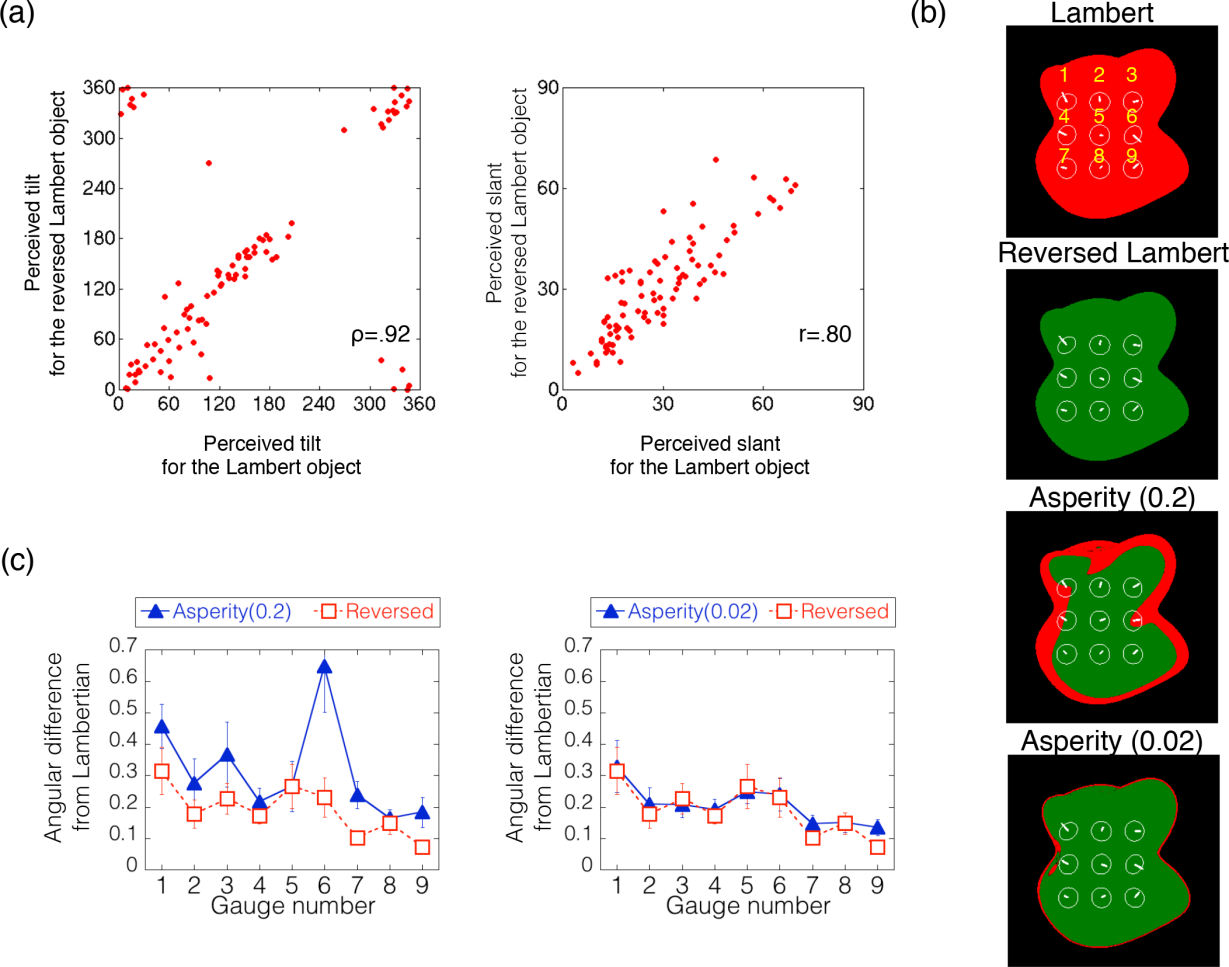
Results of Experiment 3. (a) The scatter plots of the perceived surface orientation between the normal and reversed Lambertian conditions. Left and right panels show the results for the tilt and slant, respectively. Each plot indicates the averaged judgment across trials within each observer for each gauge position. The circular correlation [29, 30] and Pearson’s correlation coefficients are shown in the tilt and slant panels, respectively. (b) The gauge positions in the stimulus. Red colors in each figure show the positions where the direction of the intensity gradient on the stimulus condition was the same as that on the Lambertian object. Green in each figure shows the positions where the direction of the intensity gradient on the stimulus condition was opposite to that on the Lambertian object. (c) The angular difference between the judgments on the two asperity conditions and the Lambertian condition. The horizontal axis indicates the gauge number, which is shown in the Fig 17(B).

Fig 17(A) shows a scatter plot of the shape estimations between the normal and reversed Lambertian objects. The correlation coefficients for tilt and slant were .92 and .80, respectively. These high correlations indicate that intensity reversal of the whole image has a negligible effect on shape perception from shading, suggesting that intensity order information relevant to shape perception is not sensitive to the direction of order.

Fig 17(C) indicates the angular difference of an asperity condition (left panel: *a* = 0.2, right panel: *a* = 0.02) from the Lambertian condition at nine gauge locations. For comparison, the angular differences of the reversed Lambertian condition are shown in the same format. When the intensity order of the asperity object was almost totally reversed (a = 0.02, Fig 17(C), right), the angular differences were as small as those for the reversed Lambertian condition. In contrast, when the intensity order of the asperity object was partially reversed (a = 0.2, Fig 17(C), right), the angular differences were higher than those for the reversed Lambertian condition. In particular, when the gauge was placed on the border where the intensity order of the asperity object changed between consistent (red in Fig 17(B)] and inconsistent (green in Fig 17(B)) with that of the Lambertian object, the angular difference was increased. We conducted a two-way repeated-measures ANOVA with the gauge location and the BRDF condition (two asperity materials and reversed Lambertian) as the within-subject variables. The two main effects and the interaction between the two variables were statistically significant (F(8,72) = 6.238, p < .0001; F(2,18) = 9.077, p = .0019; and F(16,144) = 2.381, p = .0035, respectively). The post-hoc analysis showed that the angular difference for the asperity (a = 0.2) object was statistically higher than that for the reversed Lambertian object (p = .0352, Bonferroni-corrected), while the angular difference for the asperity (a = 0.02) object was not (p = .1871, Bonferroni-corrected). These findings suggest that human shape perception is affected by local intensity order distortion of an object image even when the distortion is produced by a natural material, while it is tolerant to global intensity order reversal and relatively insensitive to intensity gradient magnitude.

**Fig 18:**
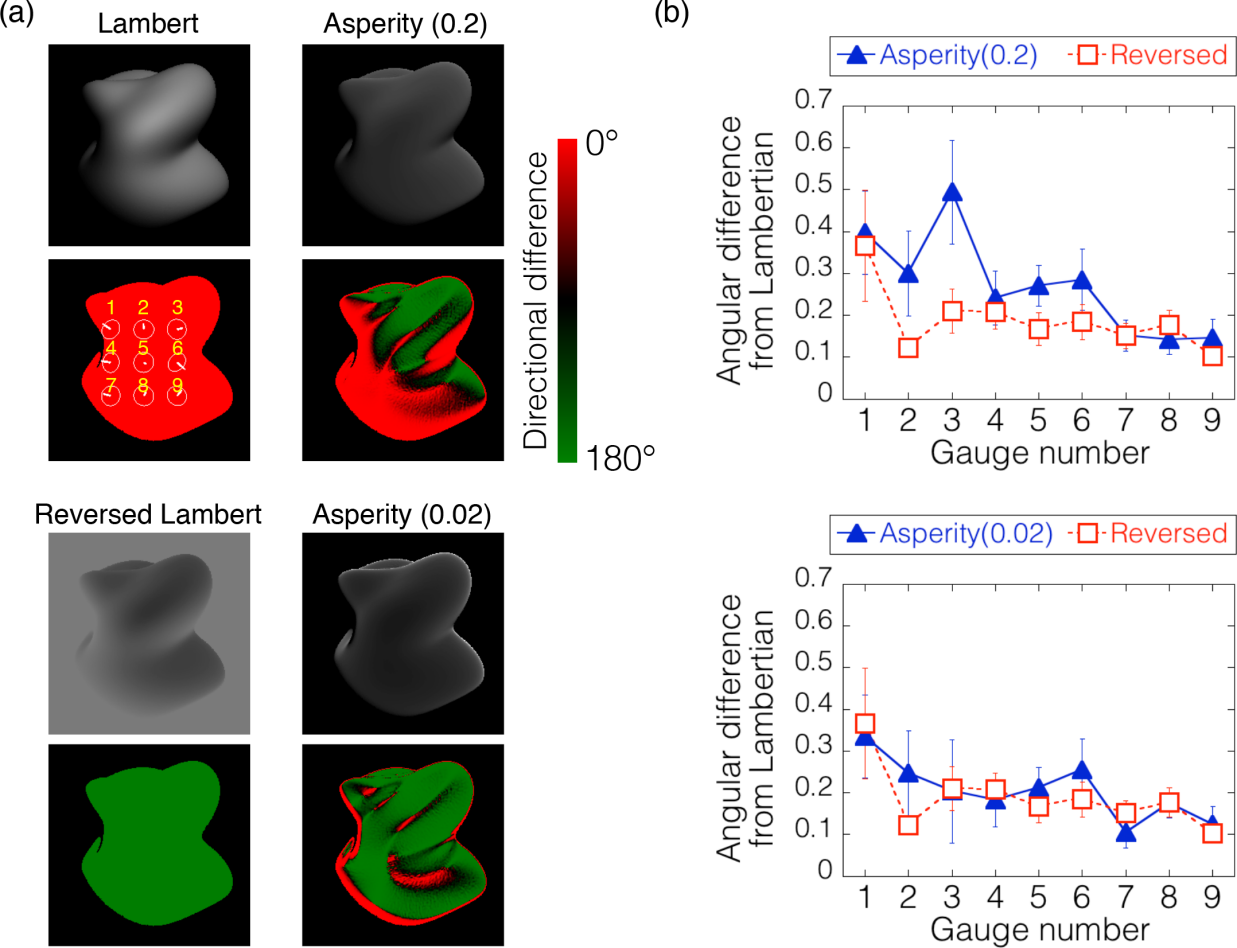
Stimuli and results of the additional experiment of Experiment 3. (a) The same stimulus set as Experiment 3 under a different lighting condition (illumination slant = 45) is used in the experiment. The directional distortion of intensity gradients from the Lambertian object image is shown in the bottom of each stimulus on the red-green axis. (b) The angular difference between the judgments on the two asperity conditions and the Lambertian condition. The horizontal axis indicates the gauge number.

In addition, to see the dependency of the illumination direction as in Experiment 2b, we additionally conducted a shape estimation experiment for a different illumination direction. When the illumination direction changes and is different from the viewing direction, the remapping constraint shown in Fig 15 is not satisfied. For that condition, the intensity order of the asperity scattering is always slightly different from that of the Lambertian image (Fig 18(A)). However, approximately speaking, when parameter *a* of the asperity scattering is relatively small (0.02), most of the intensity order in an image tends to be reversed. Results of the shape estimation experiment show that for the small *a* condition (*a* = 0.02, Fig 18(B)), the angular differences were as small as those for the reversed Lambertian condition. In contrast, when the asperity parameter is large (*a* = 0.2, Fig 18B), the angular differences were higher than those for the reversed Lambertian condition. We conducted a two-way repeated-measures ANOVA with the gauge location and the BRDF condition as the within-subject variables. The two main effects and the interaction between the two variables were statistically significant (F(8,72) = 2.654, p < .0131; F(2,18) = 8.169, p = .003; and F(16,144) = 1.743, p = .0449, respectively). The post-hoc analysis showed that the angular difference for the asperity (a = 0.2) object was statistically higher than that for the reversed Lambertian object (p = .0338, Bonferroni-corrected), while the angular difference for the asperity (a = 0.02) object was not (p = .3049, Bonferroni-corrected).

### Perception of materials and reflectance changes

In the previous section, we showed that perceived shape is sensitive to the intensity order information but not to the intensity gradient magnitude information. In this section, we will consider the perception of materials and surface reflectance properties.

Although we have seen effective modulations of perceived material by changing the intensity gradient magnitude information with no change in the intensity order information, it is hard for the visual system to estimate material only from the intensity gradient magnitude information. This is not only because of the effects of surface shape on material perception [18] but also because albedo/reflectance changes on the surface of the object affect the intensity gradient magnitude information. For example, a white patch with a steep intensity gradient on the object surface could be either a specular highlight or white paint. To distinguish between them, the visual system can use the intensity order information, since the addition of a reflectance change does, while that of a highlight does not, make the intensity order map significantly different from that of the shading pattern of an object with diffuse uniform reflectance.

When a highlight is located and/or oriented in a manner inconsistent with the shading pattern, it is perceived as an albedo change (e.g., white blob) and does not make the pattern look glossy [14–17]. While past studies proposed congruence in brightness and orientation as conditions for highlight consistency, we additionally suggest that if a white patch is a specular highlight, it does not disrupt the luminance order of the shading pattern. This means that when reducing the bright patch intensity by histogram matching to a less skewed intensity distribution or by applying a compressive tone remapping, one can smoothly erase the highlight and obtain a diffuse surface image. This should not happen if a white patch is an albedo change, since an albedo change disrupts the luminance order map. It should remain visible regardless of how the intensity gradient magnitude information is altered by the manipulation of the intensity distribution. If this hypothesis is correct, our predictions are as follows: For consistent highlights, apparent glossiness is reduced for negative skew or by compressive tone mapping, and the uniformity rating is always low. For inconsistent highlights, apparent glossiness is always low, and the uniformity rating is always high. These predictions were tested in the following two psychophysical experiments.

#### Experiment 4

**Fig 19:**
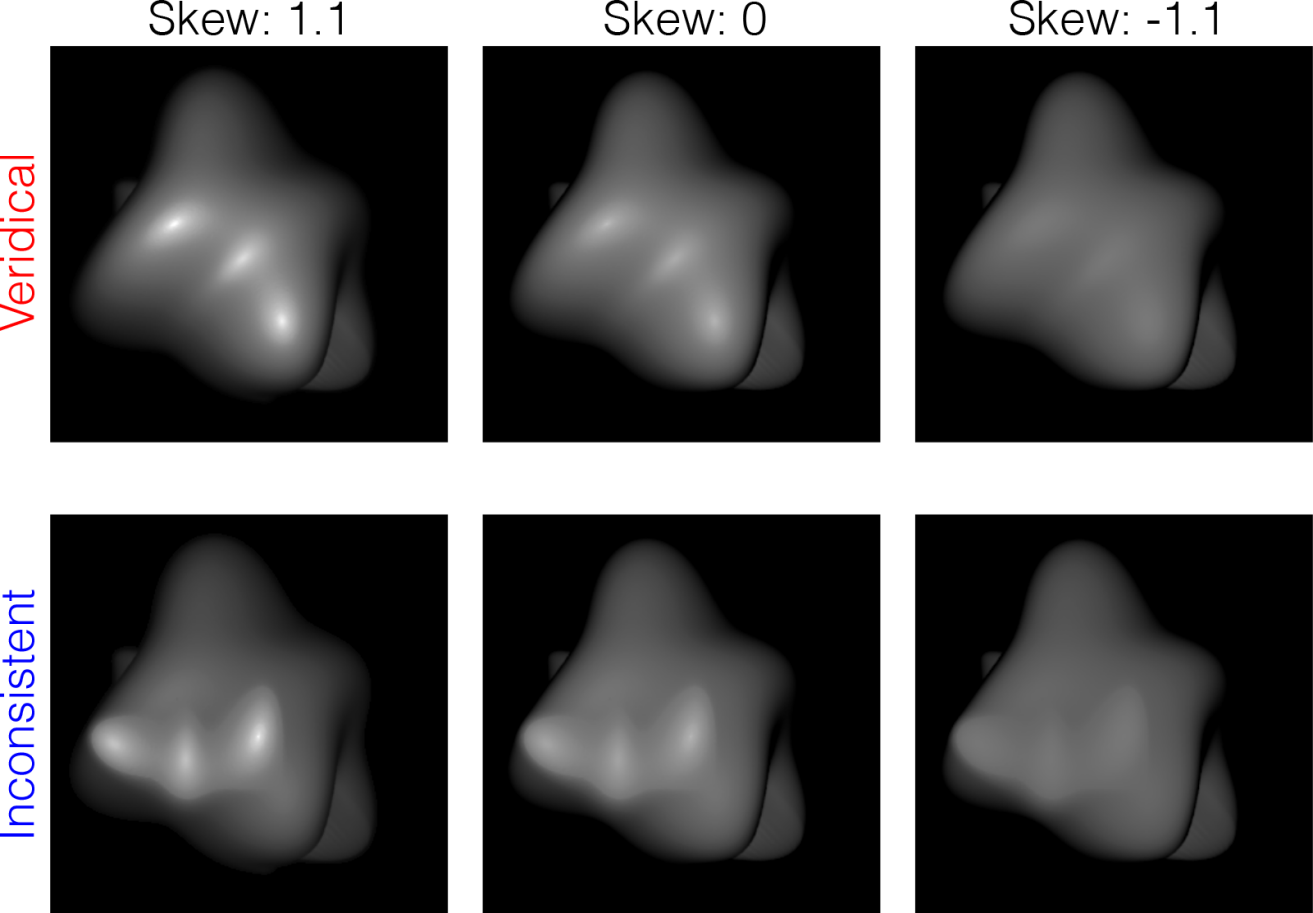
Stimuli used in Experiment 4. An object with inconsistent highlights was made by combining the diffuse pattern with the rotated and displaced specular pattern (bottom). The skewness of the intensity histogram of the object image was modulated using a standard histogram matching method. The glossy objects with veridical highlights are also shown (upper). They are the same stimuli as used in Experiment 1 (Fig 9).

In Experiment 4, to elucidate the relation between material and albedo changes of an object image, we used histogram matching to change the skewness of the intensity distribution of such object images that had either veridical or inconsistent highlights (Fig 19). We measured the perceived material (glossiness) and perceived albedo (reflectance non-uniformity) using rating tasks.

**Fig 20:**
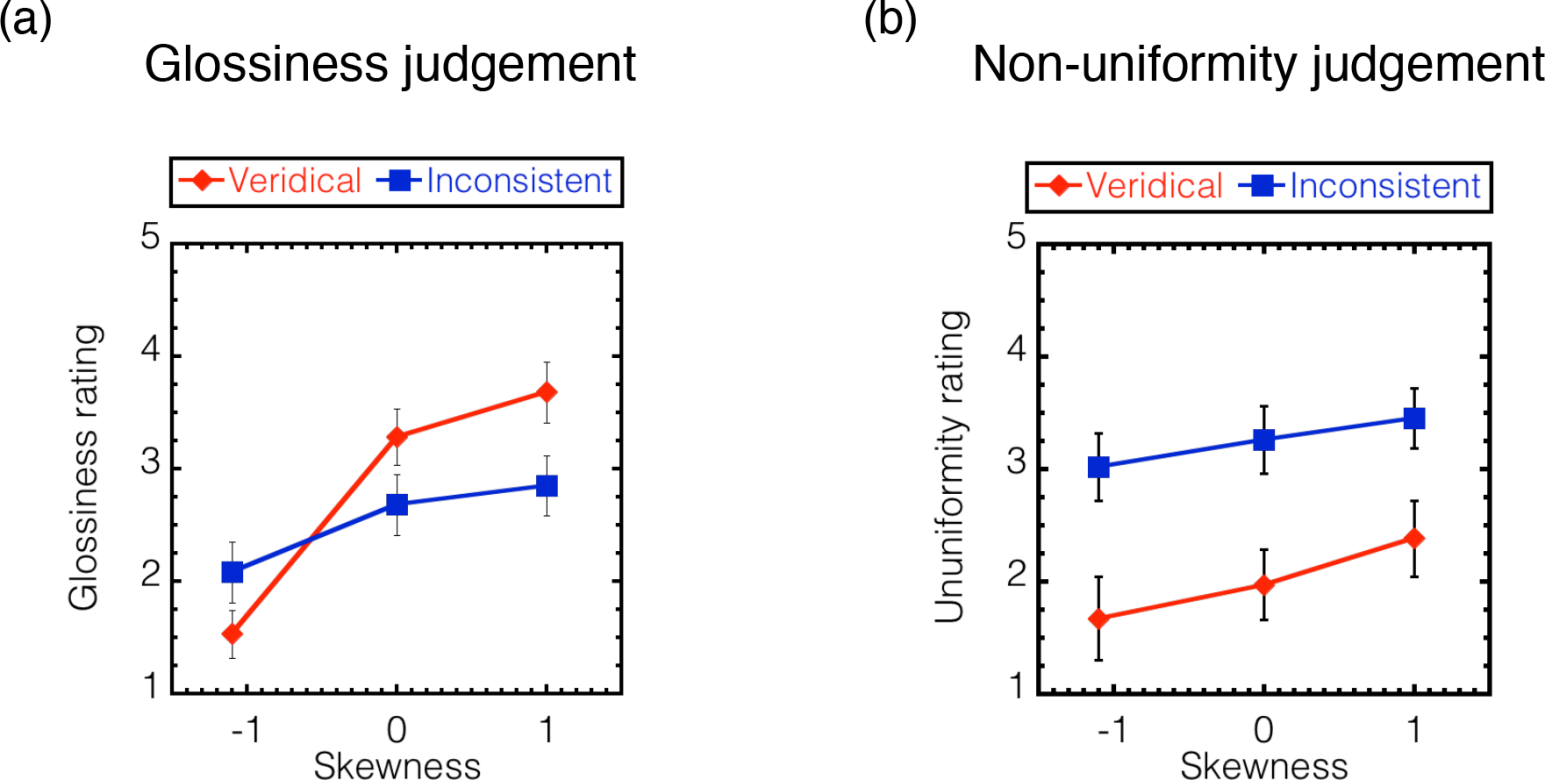
Results of Experiment 4. (a) Ratings for the glossiness judgment and (b) for the non-uniformity judgment are plotted as a function of the skewness of the intensity histogram. Stimulus conditions are shown as in the legend. Error bars indicate ± 1 SEM across observers. The ratings for the object with veridical highlights in Experiment 1 (Fig 10(A)) are plotted again in the glossiness judgment (red).

Fig 20 shows the glossiness and non-uniformity ratings, respectively, which are plotted as a function of the skewness of the intensity histogram of the object images. Each symbol indicates the averaged rating values across different observers. Different symbols denote the results under different stimulus conditions as shown in the legend. Error bars denote ± 1 SEM across observers. The glossiness ratings for the object with veridical highlights are those obtained in Experiment 1 (Fig 10(A)).

The glossiness rating shows that the objects with inconsistent highlights were perceived to be less glossy than those with veridical highlights (the same data as in Fig 10) when the luminance distribution was positively skewed. A two-way repeated-measures ANOVA with the highlight condition (veridical or inconsistent) and skewness condition (-1.1, 0, 1.1) as the within-subject variables showed that the main effect of the skewness condition and the interaction were statistically significant (F(1,7) = 34.210, p < .0001; and F(2,14) = 13.116, p = .0006, respectively), while the main effect of the highlight condition was not (F(1,7) = 0.873, p = .381). The post hoc analysis of the interaction showed that the simple main effect of the highlight condition was statistically significant for the skewness of 1.1 (F(1,7) = 8.768, p = .021). The results are consistent with previous findings [14, 15].

The non-uniformity rating shows that the objects with inconsistent highlights were judged to be less uniform than those with veridical highlights. This was the case regardless of the histogram manipulation of the image, while there was a weak trend where the non-uniformity increased with increasing skewness. We conducted a two-way repeated-measures ANOVA with the highlight condition (veridical or inconsistent) and skewness condition (-1.1, 0, 1.1) as the within-subject variables. The main effect of the highlight condition was statistically significant (F(1,7) = 11.297, p = .012), while the main effect of the skewness condition and the interaction were not (F(2,14) = 2.444, p = .123; and F(2,14) = 0.530, p = .600, respectively). The results suggest that the inconsistent highlights are more likely to be judged as reflectance changes than the veridical highlights, and this tendency is robustly observed even when histogram manipulation changes the intensity gradient magnitude information while keeping the intensity order information. In summary, the histogram manipulation of an object image affected the perceived glossiness but did not affect the perceived albedo. This finding suggests that the intensity order information of an object image is useful to separate highlights from albedo changes within the object.

#### Experiment 5

**Fig 21:**
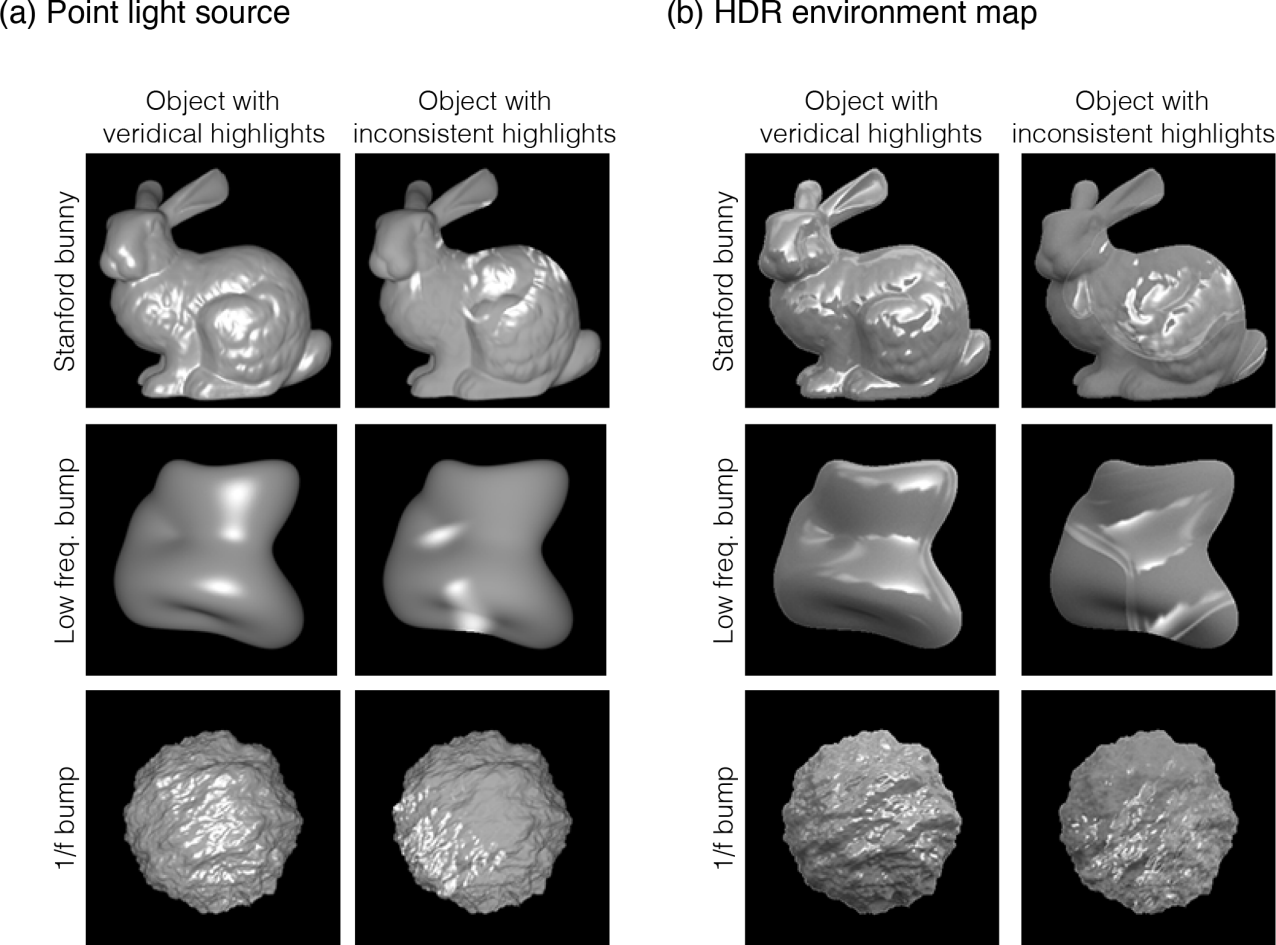
Stimulus conditions in Experiment 5. (a) Three object images (Stanford bunny, low frequency bump, and 1/f bump) were used. In addition to veridical rendering for the object, object images with inconsistent specular highlights were created. The object images were rendered under (a) the point light source or (b) the HDR environment map.

**Fig 22:**
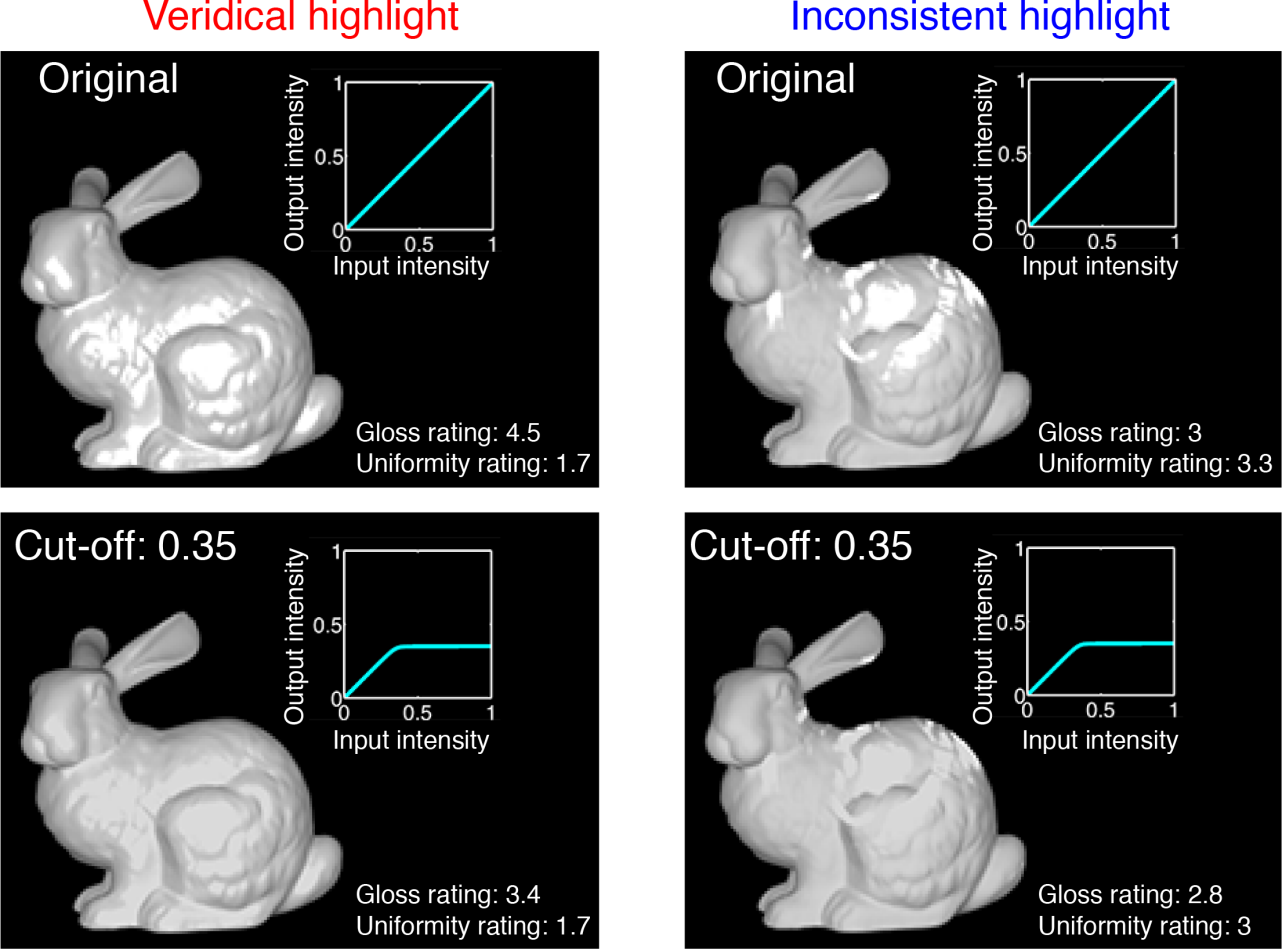
Effect of tone-remapping on the appearance of the material and albedo. We changed the cut-off intensity of compressive tone-remapping applied to several object images with veridical or inconsistent highlights.

In Experiment 4, we used simple objects under a point light source. To confirm the generality of the finding, Experiment 5 investigated the effect of luminance histograms on the perception of gloss and reflectance uniformity using more complex objects under a point light source or an HDR environment light field (Fig 21). To modulate the image intensity distribution, we used compressive tone remapping as we did when analyzing the MERL objects under the HDR illumination environment (Fig 6(B)). The compressive tone-remapping makes the histogram distribution more negatively skewed. It is mathematically equivalent to histogram matching to a more negatively skewed distribution, but, unlike the method used in Experiments 1 and 4, it does not keep the mean and standard deviation of the luminance histogram the same. Instead, the compressive tone-remapping is able to selectively dim high-intensity pixels, which are likely produced by highlights, without affecting the remaining pixels (Fig 22).

**Fig 23:**
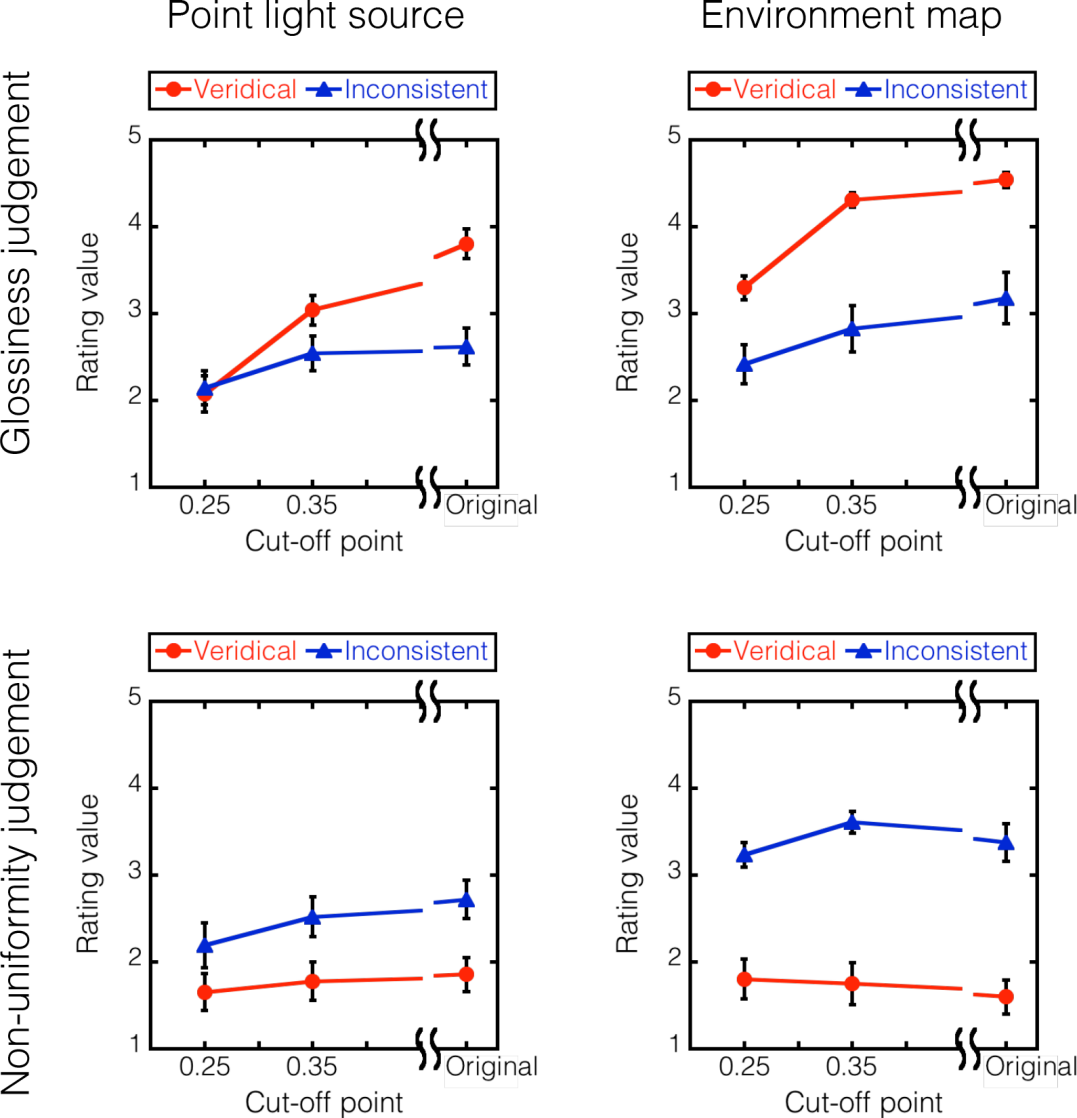
Results of Experiment 5. The rating values for the glossiness (top) and non-uniformity (bottom) judgments are plotted as a function of the cut-off point. The rating values for the object condition were pooled across observers. The column panels indicate the lighting condition (point or environment). Error bars indicate ± 1 SEM across observers.

Fig 23 showed the rating values for the glossiness judgment (red) and non-uniformity judgment (blue), each plotted as a function of the cut-off intensity of the tone mappings. Each symbol indicates the averaged rating values across different observers. Fig 23(left) shows the results for object images rendered by using the point light source, and Fig 23(right) shows those for object images rendered by Debevec's HDR environment map. Different panels indicated different highlight conditions. Since similar results were obtained across different objects, the data are pooled over the three objects.

The gloss rating for the original images with veridical highlights was relatively high and rapidly declined as the cut-off intensity was decreased, while that for the inconsistent highlight images was relatively low and only slowly declined as the cut-off intensity was decreased. Similar results were obtained for the two illumination conditions, except that the gloss rating was generally higher for the environmental illumination than for the point-light illumination.

The non-uniformity rating was low for the vertical highlight images but high for the inconsistent highlight images. In both cases, the non-uniformity rating was affected only slightly by the cut-off intensity. Similar results were obtained for the two illumination conditions, except that the non-uniformity rating for the inconsistent highlight images was slightly higher for the environmental illumination than for the point-light illumination.

In agreement with Experiment 4, the results demonstrate that compressive tone-remapping smoothly erases veridical highlights and makes the object less glossy, while it leaves inconsistent highlights visible as albedo changes regardless of the cut-off intensity. The results support the idea that the luminance order information plays a critical role in discriminating between highlights and reflectance changes. It is noteworthy that the finding was obtained not only under the point light source but also under the HDR environment map. Object images rendered using HDR environment maps have complex specular highlights, the local orientations of which could be inconsistent with shading patterns, as shown in Fig 6. However, given that such spatial non-uniformity is mainly produced in the high intensity range, it can be effectively removed by compressive tone-remapping as we saw in Fig 6.

## Discussion

The ultimate goal of our study is to comprehensively understand how human vison estimates the material property together with other object properties such as shapes, albedos, and illuminations. Our opening question was: Why does intensity histogram manipulation affect human material perception to a much greater extent than it does the perception of other physical properties. The image analyses of a variety of materials revealed that typical material changes have little effect on the intensity order information (which defines the isophote map and the direction of the intensity gradient), while they strongly affect the residual information, i.e., the magnitude of the intensity gradient. This led us to a hypothesis that the human visual system may mainly use the intensity gradient magnitude information for material perception, while it uses the intensity order information for shape perception and albedo change detection (Fig 8). The first three experiments confirmed that shape perception was affected little by the intensity gradient magnitude information but was affected strongly by the intensity order information. The last two experiments confirmed that perceptual discrimination of material-related intensity changes (veridical highlights) from albedo-related intensity changes (inconsistent highlights) is dependent on the intensity order information.

### Material processing

Numerous studies have utilized histogram-transformation methods as used in Experiment 1 to modulate the pattern of intensity histogram [1,6,8, 40–42]. As shown in our image analysis, the transformation does not disturb the intensity order information of a surface image, but it does distort the magnitude information of the intensity gradient of the image. In addition to histogram matching, compressive nonlinear tone mapping is widely used for appearance control in printing or screen display devices [4,5]. The mapping also usually retains the intensity order information of an input image. These techniques are consistent with the present finding that the modulation of the gradient magnitude information can be a diagnostic for the material appearance of the surface.

### Shape processing

If the intensity order of the image histogram of a surface is kept constant, then so is the isophote structure or the direction of the intensity gradient of the surface image. The effect of the structure of isophotes on surface shape estimation has been traditionally recognized in the context of shape-from-shading [19–27]. For instance, Koenderink & van Doorn [19–20] showed the structural relationships between the pattern of isophotes across a diffuse surface and the geometric structure of the surface. Specifically, they focused on the “Gauss map”, which is a spherical image where a surface in Euclidean space is mapped to the unit sphere. Since in simple stimulus situations (e.g., Lambertian materials under a collimated illumination) the radiance of a point in a surface only depends on the surface normal, each radiance of the surface image with an identical normal can be mapped to the same point on the sphere image. Koenderink and van Doorn showed that when a specific region of a surface image, such as a convex, concave, or saddle-shaped region, is extracted based on the local extrema of the image, its spherical image corresponds to the surface geometry in a one-to-one fashion. Then the isophotes of the Gauss map can have invariant structures related to the surface geometry, irrespective of illumination directions.

Similarly, Breton & Zucker [21] showed that under a diffuse surface illuminated by a point light source, the orientation of the intensity gradient field of the surface only depends on the geometric properties of the surface irrespective of the irradiance and the diffuse reflectance. They computed the “shading flow field” based on the orientation information and showed that the flow field can be used for shape estimation and edge classification. For instance, an attached shadow cast on a corrugated surface produces discontinuity in the continuous shading field of the surface and thus the discontinuity can be a cue for edge classification. More recently, Zucker and his colleagues introduced the idea of constructing a set of local surfaces based on the shading flow field for diffuse surfaces under any point light source [26].

Although the elegant analyses by Koenderink and van Doorn (1980) and Zucker et al. on potential shape information in intensity gradient maps assume Lambertian objects, the present findings indicate that their theories are also helpful for understanding shape perception for non-Lambertian materials.

In this regard, the contribution of the present study is to show that the effect of the intensity order information is considerably robust against material changes. Our image analysis showed that the changes in natural BRDFs (100 types) did not strongly affect the intensity order information of object images. In addition, we showed in Experiment 3 that when a specific material change distorted the intensity order information of an object image, the perceived shape was changed with the distortion. This finding is consistent with previous studies showing that shape constancy across specific materials could not be obtained [38, 39, 43, 44]. The findings suggest that human shape processing strongly relies on the intensity order information and that distortion of the information tends to cause the perceived shape’s modulation even when actual material changes produce the distortion.

Our psychophysical experiments show that keeping the intensity order constant makes the perceived shape constant (Experiment 1 and 2). In addition, when we disturb the intensity order information, the perceived shape changed with the distortion (Experiments 2 and 3). However, we emphasize that the same shape can have different intensity order maps and that different intensity order maps do not always produce different perceived shapes, due to, say, the effect of illumination differences. Our study mainly investigated material and shape perception under the conditions where an object is placed in a specific illumination environment, but as shown in the Image Analysis section (Fig 7), illumination changes can produce large distortions of the intensity gradient information. Nevertheless, identical objects under different illuminations can be perceived as similar in shape even when their intensity order information is markedly different, as shown in Experiment 2b (Fig 14). The previous studies also reported that the perception of shape and material is quite robust across different illumination contexts [45]. Hence, to recover the perceived shape from the intensity order information, the visual system has to discount the influence of the illumination field. While how it does this remains an open question, one possibility is that the occluding contour of a surface image may normalize the mid-level representation of an object obtained from the intensity order information [18]. Another possibility is that the visual system may extract some illuminant-invariant higher order differential structures from the intensity order information (cf., [26]).

A luminance-order map is far from sufficient to recover the geometrical ground truth of an object even when material information is given. Shape estimation solely from luminance-order information must introduce many ambiguities. It is obvious that the shape-from-intensity-order-map problem suffers from bas-relief ambiguity [46], since our theory concerns perception of matte (diffuse) and gloss (diffuse+specular) objects seen without light source information. In addition, in many cases, luminance-order maps must be equated between completely different shapes by adjusting material (BRDF, BSSRDF, BTF) and/or illumination parameters. Although we have not theoretically analyzed this ambiguity, this would seem to be a hard analysis, since it should consider not only geometrical optics, but also natural statistics of reflectance and illumination parameters. Furthermore, in order to understand human vision, the important issue is not only ambiguity in estimation of the ground-truth 3D structure, but also ambiguity in estimation of the perceived shape. The perceptual representation of shape is degenerated in the sense that it does not contain full detailed information about the ground truth structure, though what is perceptually represented about shape remains controversial [47]. According to our experiments, provided we preserved the luminance order map, the observers reported similar shapes. Despite enormous physical ambiguity, we found little evidence of perceptual ambiguity like that observed in the Necker cube. We think this provides an important hint about perceptual shape representations in the human brain.

Consider next the relationships of luminance-order information to orientation information that Fleming and his colleagues proposed were influential in shape estimation [23–25]. Like Zucker and his colleagues, they constructed the orientation field of a surface image. The dominant orientation of the field was determined according to the relative powers of oriented-linear filters’ outputs. They showed that the distortion of the orientation field of a surface image corresponds to the distortion of the perceived shape of the surface. In computer graphics also, the orientation field has been utilized for apparent shape editing [27]. Specifically, Vergne et al. [27] used structure tensors of a surface image to construct the orientation field and showed that the modulation of the field drastically changed the apparent shape. In addition, they showed in their statistical analysis that the orientation information of identical shapes with different materials (four types) or illuminations (four types) can be similar to each other, as in our image analysis.

Fleming and his colleagues have constructed a general framework of shape perception from image orientation information. Their investigation started from perfectly specular (mirrored) objects, and then generalized their theory to shape perception from diffuse shading, texture or contours. In contrast, our theory was based on a critical observation that luminance histogram matching affects apparent material. Since luminance histogram matching realized by monotonic luminance re-mapping can control the material appearance of an object in the range between pure matte (diffuse) and gloss (diffuse+specular), but cannot easily make perfectly specular appearances (we need non-monotonic luminance re-mapping [41]), we do not have a strong theoretical basis to assume that our theory is applicable to mirrored objects. Textured objects and line drawings are also outside our scope. Despite having a scope narrower than that of Fleming et al., our theory has more specific predictions about material and shape perception of objects within the scope of our analyses.

A critical question is what kind of directional information, i.e., a vector map modulo 180 or 360 degrees, or both, the visual system relies on. The orientation field of Fleming and his colleagues is based on a vector map modulo 180 degrees, while the present study used a vector map modulo 360 degrees to explain the perceived shape. In Experiments 2 and 3, we found that the modulation of the tone-mapping curves of a surface image changed the perceived shape of the surface, even though it only distorted the vector map modulo 360 degrees while keeping constant the vector map modulo 180 degrees. The finding suggests that at least for the class of materials we considered, shape perception is different when the orientation map is similar, but the luminance order is different, as predicted by our theory.

Fleming et al. proposed a ground theory for a wide range of monocular shape perception including cases where shape perception is similar even when luminance order is not preserved [48–50]. We also recognize that non-linear tone re-mappings that do not preserve luminance order information are able to change glossy objects into mirrored or translucent objects of similar shapes (e.g., [41]). We speculate the perceived shape distortion may depend on the spatial flow structure where the flow distortion emerges. For instance, in Experiment 3 (Fig 17) a perceived shape distortion for the asperity (a = 0.2) condition was obtained in gauge position 6 where the shading flow produces a cusp. The finding suggests that the effectiveness of the vector map modulo 360 degrees may depend on the diagnostic flow structure.

Although we show the importance of the signed intensity gradient in shape estimation, we agree that unsigned orientation measurements are also useful in shape processing. For instance, the computation based on 180 degrees would be beneficial in the estimation of specular-only images because the processing modulo 180 degrees is tolerant to the first-order modulation due to mirror reflections. Thus, the processing based on the vector map modulo either 180 or 360 degrees has benefits in some situations. This suggests a possibility that two types of processing are adopted by the visual system, and thus further studies are necessary to elucidate how human shape processing codes directional information.

While Fleming et al. [23–25] measured unsigned orientations at multiple scales, the luminance gradient computation normally has a single value at each location. Simultaneous gradient measurements at multiple spatial scales might be more beneficial, but we have not investigated this possibility yet.

In sum, although the relationship of our theory with the theory of Fleming et al. is still to be clarified, we can at least say that a luminance-order map contains richer information than that of an orientation map, and that human shape processing does not refrain from using the extra information when it is available and useful.

As for the effect of gradient magnitude, although we showed that the perceived shape is little affected by modulating the magnitude of intensity gradients, we understand that in some cases the perceived shape, especially perceived volume, can be affected by position/scale specific modulation. When intensity magnitudes are small, the surface tends to appear to have less curvature (i.e., they appear flatter) than when the magnitudes are large, even if the intensity ordering is constant. In particular, Giesel & Zaidi [51] showed that enhancing the amplitude of specific spatial frequency components increases perceived volume, although the modulation does not change the perceived tilt. It has been shown that the perceived volume (slant) of an object image is unstable, compared with its perceived tilt [52], and therefore it might be affected by several factors depending on the context in which the object is placed.

### Perception of reflectance changes

This study suggests that the computation based on two types of intensity gradient information may facilitate a comprehensive understanding of material and shape processing. In addition, we showed that this computation may also be used for the perception of reflectance changes. That is, the present study revealed that the specular-shading consistency could be judged in the shading processing as a problem of discrimination of smooth shadings from reflectance changes. It is noteworthy that in Experiments 4 and 5 the perception of albedo-uniformity for an object image with inconsistent highlights was not changed by histogram modulations (Figs 20 and 23). This finding suggests that processing based on the intensity order information may be sufficient for discriminating an object image with veridical highlights from inconsistent ones.

**Fig 24:**
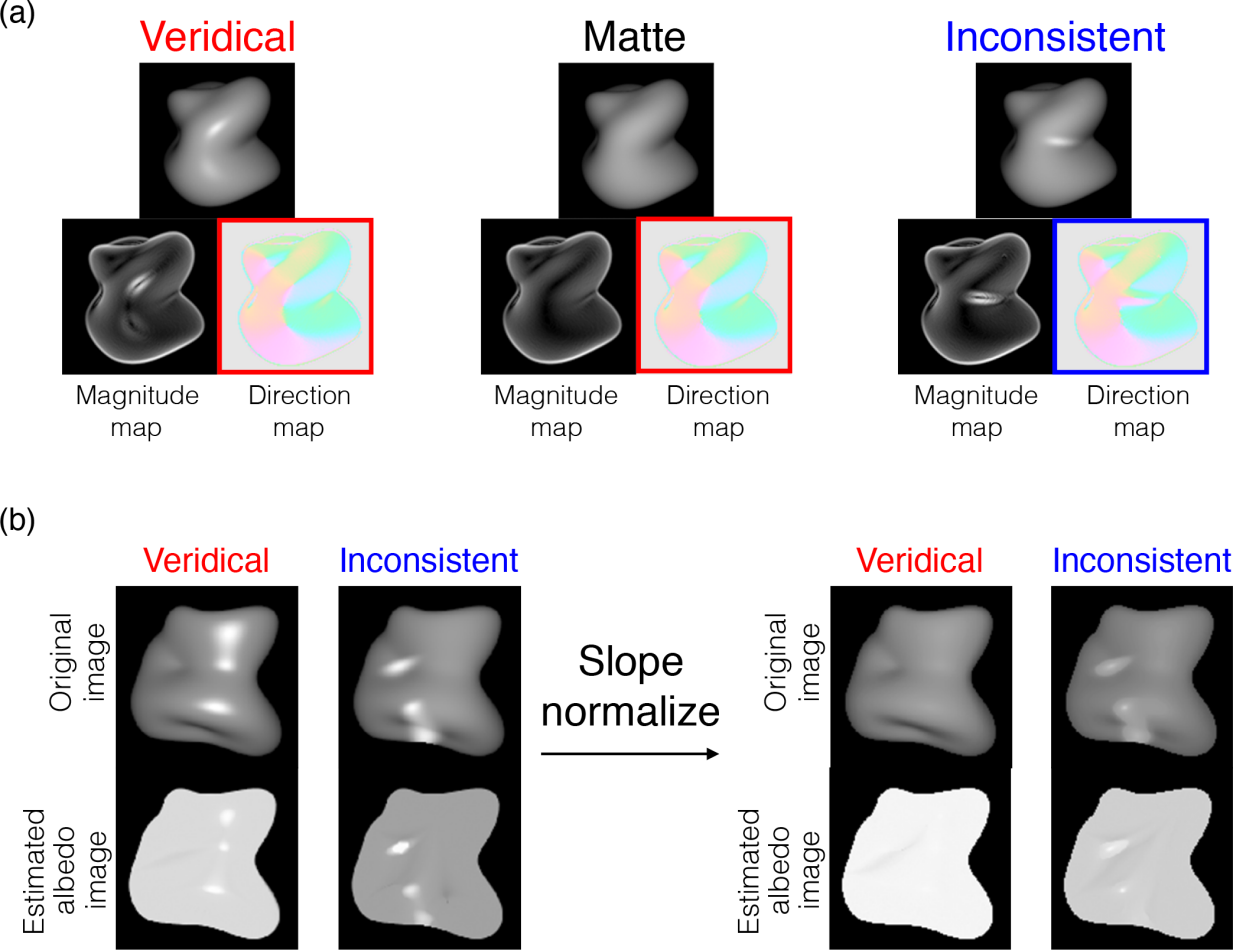
Demonstration of detecting albedo changes based on the intensity gradient. (a) Veridical highlights have little effect on the direction map, i.e., the map for the veridical highlight was similar to that for the matte one. In contrast, inconsistent highlights mark an abrupt change in the direction map. (b) When one of the intrinsic image decomposition algorithms [58] was applied to the object images with veridical and inconsistent highlights (left), even for the uniform albedo image with veridical highlights it incorrectly detects the highlights as different albedos. The estimated albedo images are shown at the bottom of the figure. In contrast, when it was applied to the slope-normalized image (right), the image with veridical highlights can be regarded as a uniform albedo.

Although the algorithms of intrinsic image decomposition in the field of computer vision can discriminate smooth shadings from reflectance changes [53–59], it is not easy for many of them to discriminate veridical specular highlights from reflectance changes such as white blobs. For instance, when one of the cutting-edge algorithms [58] is applied to object images with veridical and inconsistent highlights, even for a uniform albedo image with veridical highlights, it incorrectly detects the highlights as regions with different albedos. However, when the same algorithm is applied to a slope-normalized image (Fig 24B, right), it correctly predicts the image with veridical highlights to have a uniform albedo, while the image with inconsistent highlights to have non-uniform albedos. This observation suggests that, if the visual system has a pre-processing stage to extract a luminance-order (slope-independent) image, it can easily discriminate smooth shadings from reflectance changes, and correctly solve the highlight consistency problem.

We do not intend to dispute a previous hypothesis that the position and orientation congruence of specular highlights relative to diffuse surface shading could be critical for discrimination [13]. In terms of our hypothesis, position and orientation incongruences imply non-smooth luminance-order maps, and thus are likely to arise from albedo changes.

### Neural mechanisms

The cortical processing of intensity order information, as well as that of intensity magnitude information, remains unclear. One plausible hypothesis is that the brain decodes the direction and magnitude of the local intensity gradient from the outputs of orientation-selective filters. These filters should be located at early stages where local phase information is preserved, not at later stages where local orientation energy is represented. However, since little attention has been paid to intensity order information, we can only speculate on its cortical mechanism at present. Our brain may adopt a completely different strategy to process luminance-order information. We hope the present psychophysical findings will motivate future neurological investigations into the mechanisms of cortical processing of the intensity order and magnitude information. Specifically, it would be interesting to see which cortical areas are more sensitive to intensity order than to intensity magnitude, and vice versa. Some neurological studies have found gloss-selective neurons in the ventral stream of monkeys and common marmosets [60–62]. For instance, Nishio et al. [61] found neurons in the inferior temporal (IT) cortex of the monkey that selectively and parametrically respond to physical gloss enhancements. These neurons are likely to be more sensitive to intensity gradient magnitude information. On the other hand, object-selective neurons in the other parts of the IT cortex may be more sensitive to intensity order information.

### Conclusions

While investigating the effects of histogram transformation methods on material perception, we showed that material processing depends on detailed gradient information rather than intensity order information, such as the direction of the intensity gradient. These findings also revealed the image constraints produced by other physical properties such as albedo and shape. The present study suggested that specular-shading consistency could be judged from intensity order information, with which a specular consistency problem becomes a general shading-reflectance separation problem. In addition, our study suggests that human perception of shape from shading is sensitive to the intensity order information of an object image but not sensitive to the detailed intensity gradient information.

## Methods

### Ethics statement

All the psychophysical experiments were approved by the Ethical Committees at NTT Communication Science Laboratories and were conducted in accordance with the Declaration of Helsinki.

### Image constraints for material changes

#### Image analysis

In the analysis, we used three surface geometries, all of which were surfaces modulated in depth by Gaussian band-pass noises (Fig 3). The surface images were rendered using PBRT [63] under a point light source facing in the same direction as the viewing direction (slant = 0°), under a point light source facing in two different directions from the viewing direction (slant = 20° and 40°), or under the HDR environment map in Bernhard Vogl’s website (“Overcast day at Techgate Donaucity”, http://dativ.at/lightprobes/index.html). The size of the rendered images was 256 × 256 pix. Each rendered image was transformed to gray scale and divided into the direction and the magnitude maps of the intensity gradient using a gradient operator [64]. The size of the gradient kernel was 5 x 5 pix. Then the correlation between all pairs of material changes with the same geometry model was calculated. For the magnitude map, Pearson’s correlation coefficient was used; for the direction maps, the circular correlation was used [29,30]. The number of pixels used for the calculation of each correlation was 196,608 (256 × 256 pix × 3 surface geometries).

In one analysis, we applied a compressive tone mapping to the surfaces rendered under the HDR maps (Fig 6(B)). The compressive tone mapping was defined as follows.

$$f_{1}(L_{o})={\left[{\left(1-L_{o}\right)}^{r}+{\left(1-c\right)}^{r}\right]}^{1/r},$$ 1)

where *L*_*o*_ is the pixel intensities of the original image. The intensity ranged from 0 to 1. *c* is the cut-off intensity of the mapping, and *r* is a smoothing factor around the cut-off intensity. *r* was set to 30 in the present analysis, and c was determined in each image by subtracting 1 SD from the mean intensity. When the magnitude of the intensity gradient was close to zero after tone-mapping (threshold < 0.0002), we excluded the gradient from the correlation computation of the direction and the magnitude analyses.

### Perception of shapes

#### Experiment 1

Observers. Eight observers participated in the experiment. They were naïve to the purpose and methods of the experiment and had normal or corrected to normal visual acuity. They were paid for their participation and gave their written informed consent before the start of the experiment.

*Apparatus.* The experimental stimuli were displayed using Matlab R2013b in conjunction with the Psychophysics Toolbox 3 [65,66]. They were displayed on a calibrated 30-inch EIZO color monitor (ColorEdge CG303W) driven by an NVIDIA video card (Quadro 600) with a pixel resolution of 2560 × 1600 and a frame rate of 30 Hz. The intensity of each phosphor could be varied with 10-bit resolution. The experiment was run in a dark room.

*Stimuli.* The stimuli used in the experiment were computer-generated images (Fig 9). We made the geometry models of a bumpy sphere modulated in each surface normal direction using Gaussian band-pass noises and lit them using a point light source placed in the same direction as the viewing direction. The object images were rendered with the Ward reflection model [67]:

$$\rho (\theta_{i},\phi_{i},\theta_{o},\phi_{o})=\frac{\rho_{d}}{\pi }+\rho_{s}\frac{\exp\left[-\tan^{2}\delta /\alpha^{2}\right]}{4\pi \alpha^{2}\sqrt{\cos\theta_{i}\cos\theta_{o}}},$$ 2)

where *ρ(θi*,*φi*,*θo*,*φo)* is the surface reflection model, and *θi*, *φi*, and *θo*, *φo* are the incoming and outgoing directions, respectively. There are three parameters in the model; *ρd*, *ρs*, and *α*, where *ρd* is the diffuse reflectance of a surface, *ρs* is the energy of its specular component, and *α* is the spread of the specular lobe. The values of *ρd*, *ρs*, and *α* used in the experiment were 0.5, 0.25, and 0.25, respectively.

We applied standard linear tone mapping to the rendered images and then modulated the skewness of the intensity histogram of the object images using the histogram matching method (Fig 1). The reference distribution for the matching *h*_*3*_ was made using a Beta distribution as in [8], given by:

$$h_{3}(L_{r})=\frac{1}{B(p,q)}L_{r}^{p-1}{\left(1-L_{r}\right)}^{q-1},B(p,q)=\int_{0}^{1}L_{r}^{p-1}{\left(1-L_{r}\right)}^{q-1}dL_{r},q=10-p,$$ 3)

where *L*_*r*_ is the pixel intensity, and *p* is the parameter that modulates the amount of skewness. In the present experiment we set the values of *p* as 1.5, 5, and 8.5, which approximately correspond to skewness of -1.1, 0, and 1.1, respectively (Fig 9). The mean and SD were set to those of the original image by rescaling the Beta distribution. These stimuli were presented on the experimental monitor, the maximum luminance of which was 150.6 cd/m^2^. Each stimulus was presented in a window of 3.5 deg × 3.5 deg on the monitor.

#### Procedure

*Glossiness judgment.* In the glossiness judgment, observers rated how glossy the object image appeared to be on a 5-point scale. A rating of 1 meant that the object image appeared to be perfectly matte, while a rating value of 5 meant that the object image appeared highly glossy. Each stimulus condition was tested six times in total for each observer.

*Shape estimation using gauge probes.* In the shape estimation task, observers reported the perceived shape of the object images by setting a gauge probe with a hand-held mouse to match the apparent surface slant/tilt at nine locations per object. Following Fleming et al. [23], we simultaneously presented a pair of the same images on the monitor. One image showed the latest status of all nine probes that the observer had to match, and the other image contained only a single probe that the observer was currently adjusting (see also Fig 4A in [23]). In addition to the skew modulated objects (Fig 9), each original object with highlights was used for the control condition. Each stimulus condition was tested six times in total for each observer.

### Experiment 2a

We applied a variety of nonlinear remappings to three object images (Fig 11). The remapping function was defined as follows.

$$f_{4}(L_{o})=s_{1}(L_{o}-m)+s_{2}\sin(\omega (L_{o}-m)+\phi )+m,$$ 4)

where *m* is the mean intensity, *s*_*1*_ is the slope of the remapping function, *s*_*2*_ is the amplitude of a sinusoidal modulation, ω is the angular velocity of the modulation, andφis its phase. Specifically, we made fifteen remapping curves by adding a sinusoidal modulation with one of five different amplitudes (*s*_*2*_ = 0, 0.015, 0.065, 0.115, or 0.165) to a linear tone remapping function with one of three different slopes (*s*_*1*_ = 0.5, 1, or 2). The ω and φ in the experiment were the constant values of 2.857π and π, respectively. When the slope was the steepest (2), the remapping curve always monotonically increased. This implies that the intensity order of the original image was not disrupted even by the largest modulation. When the slope was midway between steep and gentle (1), the remapping curve was non-monotonic, and the intensity order of the original image was disrupted when *a*_*1*_ was 0.115 or 0.165. When the slope was gentle (0.5), the intensity order was disrupted when *a*_*1*_ was 0.065, 0.115 or 0.165. It should be noted that our manipulation did not change the orientation (modulo 180 degrees) map of the image. The geometric models we used in the experiment were two bumpy spheres (the displacement in the normal direction of the surface of each sphere was given by a coarse or fine Gaussian band-pass noise; Fig 11, bottom left and bottom center), and a cylinder (Fig 11, bottom right). Each model was lit by a point light source from the camera direction.

Eight observers were asked to estimate the perceived shape of the objects by setting a gauge probe with the matching apparent surface slant/tilt. The position of the nine gauge probes is shown in Fig 11. Each of the 45 stimuli (3 objects x 15 tone-mapping types including the original image) was tested three times for each observer. In addition, to confirm the performance stability of the gauge task, the gauge matching for the original images of the three objects was again conducted three times in a different session. These data were used for the baseline and showed as no shape changes in Fig 12. The other methods were the same as in Experiment 1.

### Experiment 2b

In the experiment, we applied a variety of non-linear remappings, as in Experiment 2a, on several object images rendered under a point light source place in the same direction as the viewing one or in the upper-right direction for the object where the illumination slant was 45° and the illumination tilt was 30°. Ten observers were asked to estimate the perceived shape of the objects by setting a gauge probe with the matching apparent surface slant/tilt. The position of the six gauge probes is shown in Fig 13. Each of the 10 stimuli (2 illumination direction x 5 tone-mapping types including the original image) was tested ten times for each observer. The other methods were the same as those used in Experiment 2a.

### Experiment 3

The geometric model was a bumpy object with low spatial frequency bumps (Object 4) (Fig 16). The model was lit by a point light source from the camera direction. The reflection model used in the experiment was the Lambertian or asperity material [38, 39]. The output intensities on the models were determined as follows:

$$L_{l}=\frac{\rho_{d}}{\pi }l_{l}(I\cdot N),$$ 5)

$$L_{a}=\frac{a}{\pi [a+(I\cdot N)(J\cdot N)]}l_{a}(I\cdot N),$$ 6)

where *L*_*l*_ and *L*_*a*_ are the intensity of the Lambertian and asperity materials, respectively. *I*, and *J* are the incident and reflected angles, respectively. *N* is the surface normal, *l* is the intensity of the light source, and *a* is the asperity parameter known as the edge brightening factor. When the incident and reflected angles are the same, which is true under the lighting condition we used, the intensity of the asperity material, *L*_*a*_, can be described as a function of the intensity of the Lambertian material as follows:

$$L_{a}=\frac{al_{a}L_{l}}{a\rho_{d}l_{l}+\frac{\pi^{2}L_{l}^{2}}{\rho_{d}l_{l}}}$$ 7)

This could be regarded as an intensity remapping function from Lambertian to asperity materials, the shape of which is dependent on parameter *a* (Fig 15). As the *L*_*l*_ increases, the remapping function first rises and then falls after a transition point. The transition point (peak) shifts towards the lower range with a decrease in *a*. When *a* is moderately small (0.2), the intensity order is preserved in the lower range of Lambertian pixel intensity, while reversed in the higher range. When *a* is even smaller (0.02), the intensity order is reversed in most of the intensity range. We rendered asperity objects using these two values of *a* (Fig 16). In the experiment, the parameters *l*_*l*_, and *ρ*_*d*_ were π and 0.6, respectively. The parameter l_a_ was π under the asperity(a = 0.2) condition, and 4π under the asperity(a = 0.02) condition. In addition to a Lambertian object and two asperity objects, we used an object the intensity of which was completely reversed from that of the Lambertian object (Fig 16).

Ten observers participated in Experiment 3. The observers were asked to estimate the perceived shape of the objects by setting a gauge probe. The other methods were the same as those used in Experiment 2.

The same ten observers in Experiment 3 participated in one additional experiment. In the experiment, the geometric model was lit by a point light source in the upper-right direction for the object where the illumination slant was 45° and the illumination tilt was 30° (Fig 18). The same four reflection models and nine gauge probes as those in Experiment 3 were used. The observers were asked to estimate the perceived shape of the objects by setting a gauge probe.

### Perception of reflectance changes and materials

#### Experiment 4

Unless otherwise noted, the methods were the same as those used in the rating experiment of Experiment 1.

To make object images with inconsistent specular highlights (Fig 19), in which the highlights are incongruent in position and orientation with the diffuse shading component, we decomposed original glossy object images illuminated by a point-light source into diffuse and specular patterns and recombined the diffuse pattern with a rotated and displaced version of the specular pattern. We then modulated the image intensity modulation by histogram-matching to a Beta function (Eq 3) with skewness parameter *p* set to -1.1, 0 or +1.1. We also used matte images with Lambertian reflection (i.e., *ρs* = 0 in Eq (2)).

The observers were asked to rate glossiness and non-uniformity. In the glossiness judgment, observers rated how glossy the object image appeared on a 5-point scale. In the non-uniformity judgment, observers rated the degree of reflectance non-uniformity of the object images on a 5-point scale. A rating value of 1 on the scale meant that the object image appeared to have completely uniform albedo; a rating value of 5 meant that the object image appeared to have many dark stains or paint marks on a uniform albedo surface. The same eight observers in Experiment 1 participated this experiment.

#### Experiment 5

We used three types of object models: the Stanford bunny (Fig 21(A) and 21(B) Stanford bunny) [68], a bumpy object with low spatial frequency bumps (Fig 21(A) and 21(B), low freq. bump), and a sphere with 1/f noise bumps (Fig 21(A) and 21(B), 1/f bump). We lit our models using a point light source facing the same direction as the viewing direction or using one of Debevec's HDR environment maps, “pisa” [69]. The object images were rendered with the Ward reflection model (Eq (2)] using Mitsuba’s physically based renderer [70]. The values of parameters *ρd* and *ρs* used in the experiment were 0.5 and 0.15, respectively. The value of parameter *α* for object images under the HDR environment map was 0.1.

In addition to veridically rendered specular images (Fig 21(A) and 21(B), left panel), we made object images with inconsistent specular highlights (Fig 21(A) and 21(B), right panel) by combining the diffuse pattern with a rotated and displaced version of the specular pattern.

For the 18 object images [Object (bunny, low freq., and 1/f noise) × Lighting (point and environment) × Highlight-consistency (consistent and two types of inconsistent images)], we modulated the pixel intensity distribution by changing the cut-off intensity of Eq (1). In the experiment, the values of *c* were 0.25, 0.35, or 1. The value of *n* was fixed at 30.

Two rating tasks were conducted: glossiness and non-uniformity, as in Experiment 4. Eight observers participated in Experiment 5.

## Supporting information

S1 Dataset Individual data of all psychophysical experiments. (ZIP)
